# Filtering effect of temporal niche fluctuation and amplitude of environmental variations on the trait-related flowering patterns: lesson from sub-Mediterranean grasslands

**DOI:** 10.1038/s41598-017-12226-5

**Published:** 2017-09-20

**Authors:** Andrea Catorci, Karina Piermarteri, Károly Penksza, Judit Házi, Federico Maria Tardella

**Affiliations:** 1 School of Biosciences and Veterinary Medicine, University of Camerino, Via Pontoni 5, 62032 Camerino, Italy; 2 School of Advanced Studies, University of Camerino, Via Lili 55, 62032 Camerino, Italy; 3 Szent István University, Faculty of Agricultural and Environmental Sciences, Institute of Botany and Ecophysiology, Páter K. st.1, Gödöllő, 2100 Hungary

## Abstract

Timing of flowering is a critical component of community assembly, but how plant traits respond to heterogeneity of resources has been identified mostly through observations of spatial variations. Thus, we performed a trait-based phenological study in sub-Mediterranean grasslands to assess the importance of temporal variation of resources in the species assemblage processes. We found that early flowering species have traits allowing for slow resource acquisition and storage but rapid growth rate. Instead, mid- and late-flowering species exhibited sets of strategies devoted to minimizing water loss by evapotranspiration or aimed at maximizing the species’ competitive ability, thanks to slow growth rate and more efficient resource acquisition, conservation and use. Our findings were consistent with the fluctuation niche theory. We observed that the amplitude of the environmental fluctuations influences the type and number of strategies positively filtered by the system. In fact, in the most productive grasslands, we observed the highest number of indicator trait states reflecting strategies devoted to the storage of resources and competition for light. Results seem also indicate that temporal variation of resources plays a role in trait differentiation and richness within a plant community, filtering traits composition of grasslands in the same direction, as formerly proved for spatial heterogeneity of resources.

## Introduction

The timing of flowering is a critical component of community assembly^1,2^. Flowering phenology affects the composition of plant communities through its effect on species interactions, for example, by competition for resources^3^ and, in turn, is affected by the plant community structure and above-ground biomass^4,5^. Moreover, flower production is resource-intensive^6,7^ and, as postulated by the phenological “mid-domain hypothesis”, should tend to favor reproduction during times of low environmental stress^8^. In fact, the mid-domain effect is a generated peak in richness at the mid-point of a bounded n-dimensional domain and can be applied to a one-dimensional temporal gradient, where randomly placed flowering periods will tend to overlap most frequently at the mid-domain, in this case, the middle of the growing season^8^. Craine *et al*.^9^ argued that if flowering time is critical to competition for resources or avoidance of stress, it could be considered a part of a larger plant strategy that incorporates other functional traits. In fact, it was postulated that the flowering pattern in grassland ecosystems is driven by functional differentiation of species inside the community, so that each phenological phase tends to be linked to a specific set of functional traits^10^. Wolkovich & Cleland^11^ observed that early and late flowering species exhibit different trait-based resource acquisition strategies; the former have a rapid-growth and low-investment strategy, while the latter have more efficient trait-based access to acquisition, transport and use of resources. It was also argued that some species may exhibit a rapid-growth and low-investment strategy in the late season, as well^12^. Catorci *et al*.^10,13^ proved that subordinate and accidental species need functional strategies allowing them to flower before or after dominant species, or to share the same period through a different type of space occupation, in order to limit competition with dominant species, and that enable tolerance to environmental stresses, which change throughout the growing season.

Therefore, temporal dimension is an important factor in determining species assemblages. However, how plant traits respond to variability of resources has been identified mostly through observations of spatial variations in trait values across environmental gradients^14–16^ or in different disturbance types and intensities^15,17,18^. Instead, since resources are heterogeneously distributed in both space and time^16^, plants likely need adaptations to cope with both spatial and temporal patterns of resource availability and to face limiting environmental conditions at different times (e.g. seasonal variations in temperature and humidity^19^). Therefore, there is a need for studies that examine phenological patterns at the plant community level and focus on multiple plant traits, in order to better understand the correlation between abiotic/biotic constraints and phenology^5^. Moreover, field observations of trait-based phenological events in habitats with different temporal variations of environmental conditions may also test the relevance of the fluctuation niche theory^20^. This theory postulates that the heterogeneity resulting from temporal fluctuations of resources can be considered another dimension of the niche. Similarly to other niche dimensions, it is expected to correspond to plant attributes variability^20^. The ecological rationale for enhanced coexistence with increasing fluctuations is based first on the different growth response of species to resources availability. If resources availability fluctuates, the temporal advantage of one species becomes balanced by the advantage of the other species at another time, but if resources availability remains constant, one of the species is likely to competitively exclude the other. Secondly, coexistence is ensured by the ability of species to tolerate interpulse periods^20^.

From a phenological point of view, if fluctuations are important in determining species assemblages, we might expect that: (i) flowering phenology is characterized by patterns of traits fitting the life cycle to the variation of environmental conditions; (ii) these patterns are based on a common trait set regardless of the type of plant community, at least under a homogeneous macro-climate.

Wishing to better understand the relationship among seasonal fluctuation of soil humidity, temperature and canopy height, and the variation of functional traits related to the species flowering patterns, we performed a trait-based phenological study in a sub-Mediterranean pastoral system of central Italy. The sub-Mediterranean bioclimate is characterized by large seasonal variations in temperature and rainfall, resulting in a peak of growth rate and phytomass production in late spring followed by a drought stress period in mid-late summer^21,22^. We studied three plant communities marked by a productivity gradient related to the different slope aspect and soil depth of their locations^23^. We hypothesized that temporal variations of environmental conditions shaped a common trait-related flowering pattern, driving the temporal niche partitioning. We expected that: (1) species flowering in mid-season, that is, in the period with higher availability of resources, are equipped with traits tied to competition, allowing for efficient acquisition and use of resources, such as runner, rhizome, summer green and persistent green leaves; (2) early and late-flowering species exhibit traits linked to a rapid-growth strategy and to stress tolerance ability, respectively, such as bulbs, spring green leaves, annual life form, succulent leaves, tap roots and prostrate growth form. Moreover, since the degree of resource fluctuation may change due to the duration of different stresses and the frequency of resource pulses^24^, we also hypothesized that: (3) context-dependent variation (environmental conditions and site management) may induce specific functional responses that partially could modify the general pattern; (4) the differences in environmental fluctuations among the three studied communities affect in different ways the trait-related blooming variability and the associated temporal niche segregation.

## Results

Trends of temperature and precipitation highlight a drought stress period in the late summer (Supplementary Fig. S1). The seasonal trend of soil temperature, soil relative humidity and canopy height indicate context-dependent differences in climate-related features and temporal shifts (Supplementary Fig. S2). Soil temperature and humidity had the highest average values on south-facing slopes and flat lands, respectively, throughout the entire growing season. Supplementary Table S1 reports the basic descriptive statistics of *F*_*x,t*_ values (namely, the mean proportions of flowering shoots occurring in plot *x* at time *t*, for species sharing a trait state), for each plant community at each observation time.

Table 1 shows the Indicator species analysis (ISA) results. Species blooming in the first half of the spring (observation times 2–4) exhibited a set of eight indicator traits, mostly common to the three communities. Instead, in the central phase of the growing season (observation times 4–7) flat areas showed the highest number of indicator traits. Some trait states were indicators throughout a wider time range (from early spring to mid-late summer), such as tubers in the three communities, annual and biennial life span in southerly ones, and persistent tap root and pleiocorm in flat land community (Table 1).

**Table 1:** Indicator trait states of observation times (single times or groups of two or three times in which the observed indicator value has the maximum value) in the three grassland communities located on north-facing slopes, south-facing slopes, and flat land.

Trait states	Topographic position of grassland communities					
	South-facing slope		North-facing slope		Flat land	
	Time/times group with maximum IV	IV	Time/times group with maximum IV	IV	Time/times group with maximum IV	IV
*Life span*						
Annual	3, 5, 6	0.529	3, 5, 6	0.489	5, 6, 7	0.680
Biennal	2, 6, 9	0.296	4, 5, 6	0.248	5	0.310
*Vegetative propagation*						
Bulbils		—	3, 4, 5	0.480	5	0.458
Runner, runner like rhizome	4, 6, 7	0.561	—	—	5, 6, 7	0.762
Rhizome	—	—	—	—	4, 5, 7	0.596
*Storage organs*						
Absent	—	—	3, 5, 7	0.563	4, 6, 7	0.629
Bulb	3, 4	0.473	—	—	5	0.449
Tuber	3, 5, 6	0.567	3, 5, 6	0.523	1, 3, 7	0.714
Persistent tap root	—	—	—	—	3, 5, 6	0.602
*Leaf persistence*						
Persistent green	—	—	—	—	4, 5, 7	0.635
Spring green	3, 4	0.421	3, 4, 5	0.629	1, 3, 4	0.937
Overwintering green	2, 3, 4	0.593	3, 4	0.340	4, 5, 6	0.648
*Leaf anatomy*						
Succulent	7, 8	0.496	—	—	—	—
Succulent/ hygromorphic	3	0.450	3, 4, 5	0.656	—	—
Hygromorphic	—	—	—	—	5	0.727
Mesomorphic/hygromorphic	3, 4	0.904	4, 5	0.555	4, 5, 6	0.694
*Horizontal space occupation*						
Absent	—	—	—	—	4, 5, 6	0.669
Caespitose	4, 5, 6	0.699	5, 6, 7	0.762	4, 6, 7	0.723
Pleiocorm	—	—	—	—	3, 5, 6	0.738
Reptant	—	—	—	—	4, 5, 7	0.404
Prostrate	—	—	—	—	5, 7	0.424
Rosettes with narrow, long leaves	3, 4	0.576	—	—	1, 5	0.780
*Vertical space occupation*						
Grass	6, 7, 9	0.604	—	—	4, 6, 7	0.707
Rosette forbs	—	—	—	—	2, 3, 4	0.594
Hemirosulate upright forb	—	—	—	—	4, 5, 6	0.613
Erosulate upright forb	—	—	—	—	4, 6, 7	0.613
Prostrate forb	5, 6, 9	0.680	5, 6, 7	0.477	5, 7	0.355
*Plant height (cm)*						
21–40	—	—	—	—	4, 6, 7	0.642
41–60	6, 7	0.615	—	—	4, 5, 7	0.619
61–80	—	—	—	—	4, 5, 6	0.542

Indicator trait states were identified by indicator species analysis performed on the “relevés x trait states (mean flowering proportion)” matrix for each plant community. Only significant indicator values (*P* < 0.05) whose relative abundance value was higher than or equal to 0.60 and whose relative frequency value was higher than or equal to 0.25 are shown. IV - Observed indicator value; 1–4 Apr; 2–18 Apr; 3–2 May; 4–16 May; 5–30 May; 6–13 Jun; 7–30 Jun; 8–20 Jul; 9–5 Aug.

In the Redundancy analysis (RDA) model of south-facing slopes, predictors explained 53.6% (*adj. R* ^2^, *P* = 0.001) of the total variance, 43.2% linked uniquely to time (*P* = 0.001), and 10.5% to time-related environmental variability, while environmental variables alone did not explain a significant portion of the total variance (*adj. R* ^2^ = −0.10; *P* = 0.795). The first three RDA axes accounted for 30.6, 8.6, and 6.6% of the total variance (55.5, 15.6, and 11.9% of the constrained variance). Regarding north-facing slopes, predictors explained 49.0% (*P* = 0.001) of the total variance, 30.6% linked to time (*P* = 0.001), 0.36% to environmental variables (*P* = 0.002) and 18.0% to time-related environmental variability. The first three RDA axes explained 34.0, 6.2, and 3.3% of the total variation (67.1, 12.1, and 6.7% of the constrained variance). Predictors explained 57.8% (*P* = 0.001) of the total variance of the flowering proportion of trait states on flat lands, 30.5% (*P* = 0.001) exclusively linked to time, 0.43% (*P* = 0.001) to environmental conditions, and 26.9% to the time-related environmental variability. The first three RDA axes explained 30.1, 12.6, and 5.9% of the total variability (50.8, 21.2, and 10.0% of the constrained variance).

**Figure 1:**
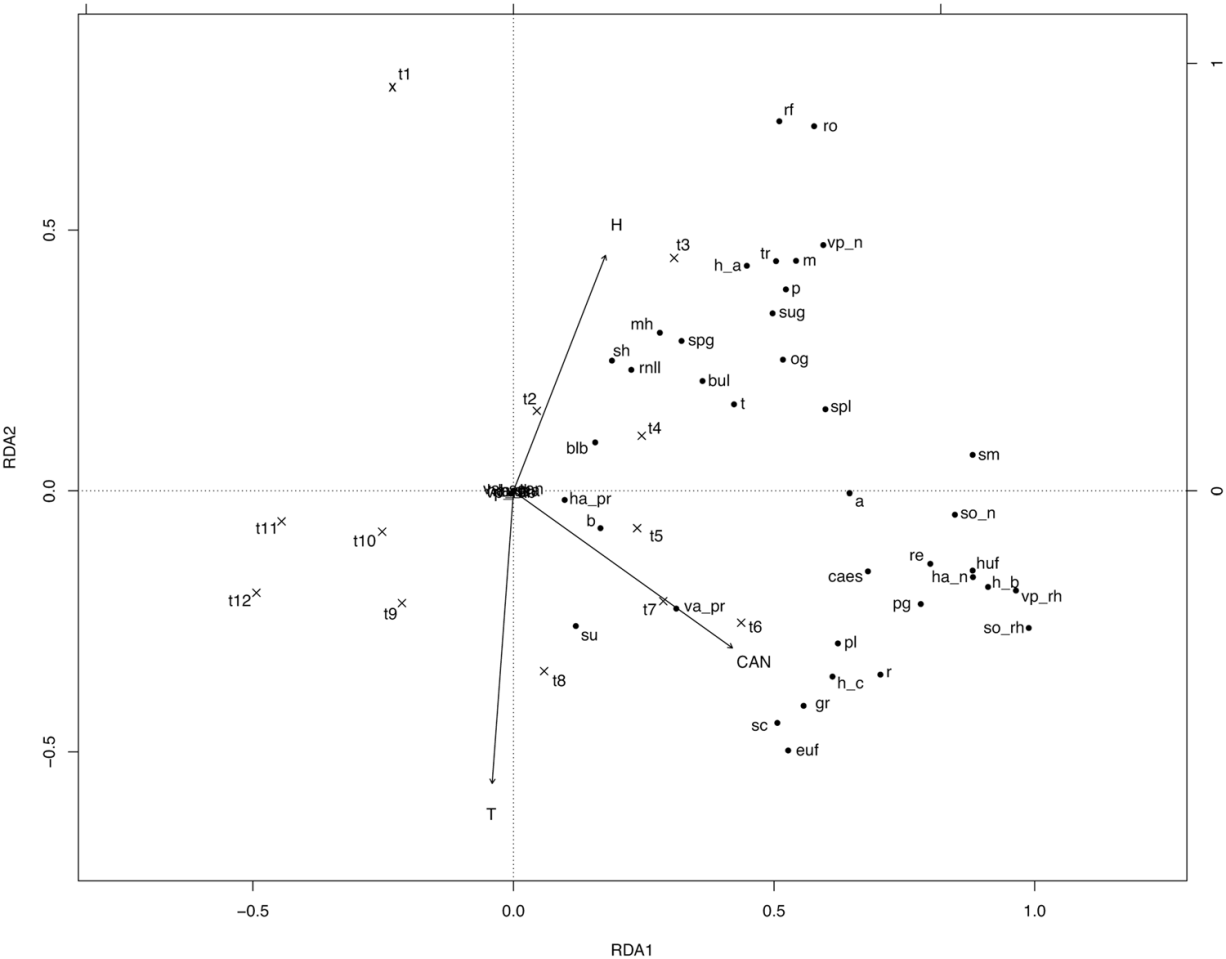
Canonical redundancy analysis ordination graph for the south-facing slope area data set, performed on the matrix “plots-by-*F*_*x,t*_ (mean proportion of flowering shoots occurring in plot *x* at time *t*, for species sharing a trait state)”, constrained by soil temperature (T), soil relative humidity (H), canopy height (CAN) and observation time (t1–4 Apr; t2–18 Apr; t3–2 May; t4–16 May; t5–30 May; t6–13 Jun; t7–30 Jun; t8–20 Jul; t9–5 Aug; t10–19 Aug; t11–6 Sep; t12–21 Oct). Legend. a annual; b biennial; blb bulbils; bul bulb; caes caespitose; euf erosulate upright forb; gr grass; h plant height (h_a < 20 cm; h_b 21–40 cm; h_c 41–60 cm; h_d 61–80 cm; h_e 81–100 cm); ha horizontal space occupation; huf hemirosulate upright forb; hy hygromorphic leaves; m mesomorphic leaves; mh mesomorphic/hygromorphic leaves; n absence of the trait; og overwintering green leaves; p perennial; pg persistent green leaves; pl pleiocorm; pr prostrate; r runner/runner-like rhizome; re reptant; rf rosette forb; rh rhizome; rnll rosettes with narrow, long leaves; ro rosulate; sc scleromorphic leaves; sh scleromorphic/hygromorphic leaves; sm scleromorphic/mesomorphic leaves; so belowground storage organ; spg spring green leaves; spl root, tuber or bulb splitter; sh succulent/hygromorphic leaves; su succulent leaves; sug summer green leaves; t tuber; tr persistent tap root; va vertical space occupation; vp vegetative propagation.

**Figure 2:**
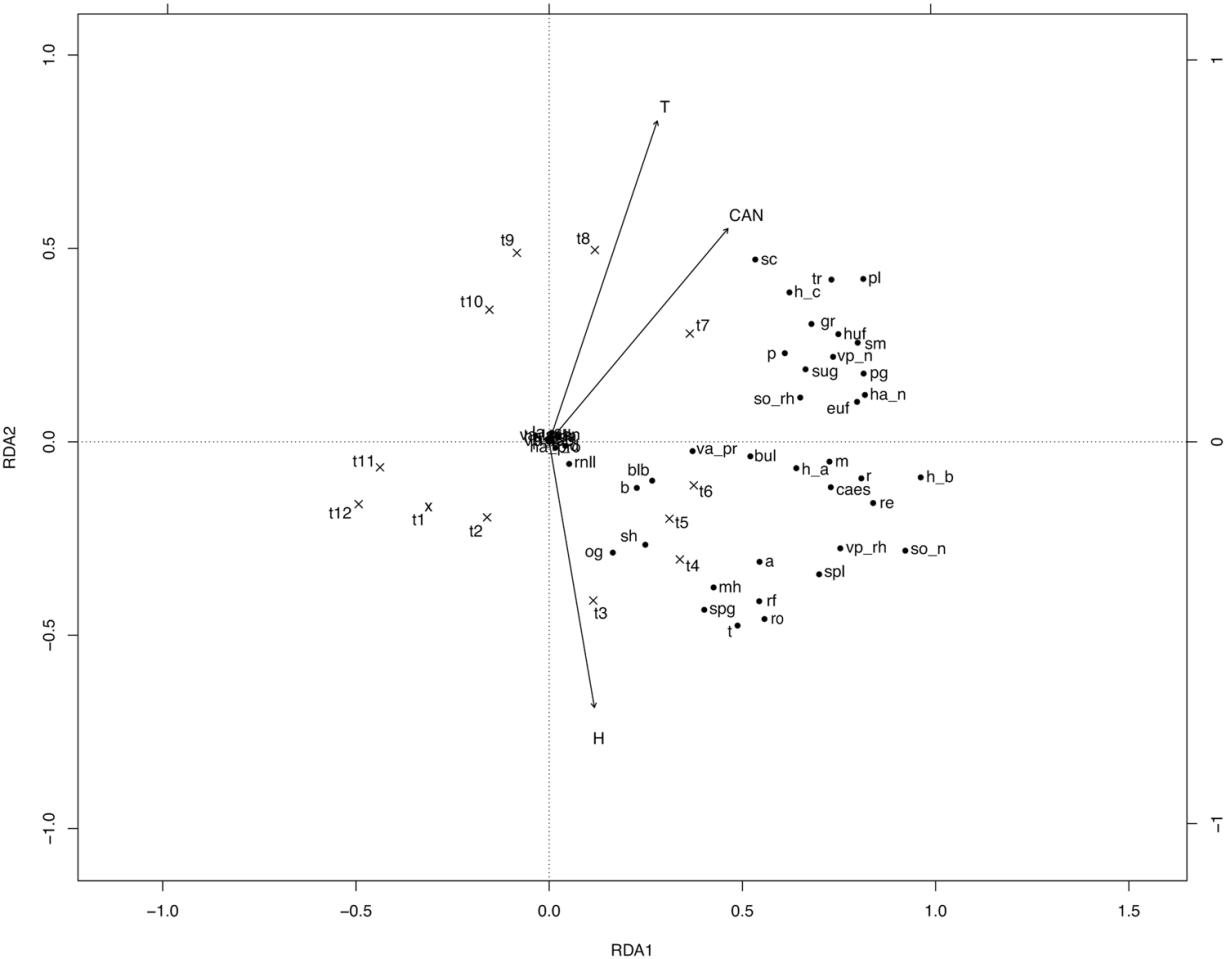
Canonical redundancy analysis ordination graph for the north-facing slope area data set, performed on the matrix “plots-by-*F*_*x,t*_ (mean proportion of flowering shoots occurring in plot *x* at time *t*, for species sharing a trait state)”, constrained by soil temperature (T), soil relative humidity (H), canopy height (CAN) and observation time (t1–4 Apr; t2–18 Apr; t3–2 May; t4–16 May; t5–30 May; t6–13 Jun; t7–30 Jun; t8–20 Jul; t9–5 Aug; t10–19 Aug; t11–6 Sep; t12–21 Oct). The legend is the same as that of Fig. 1.

**Figure 3:**
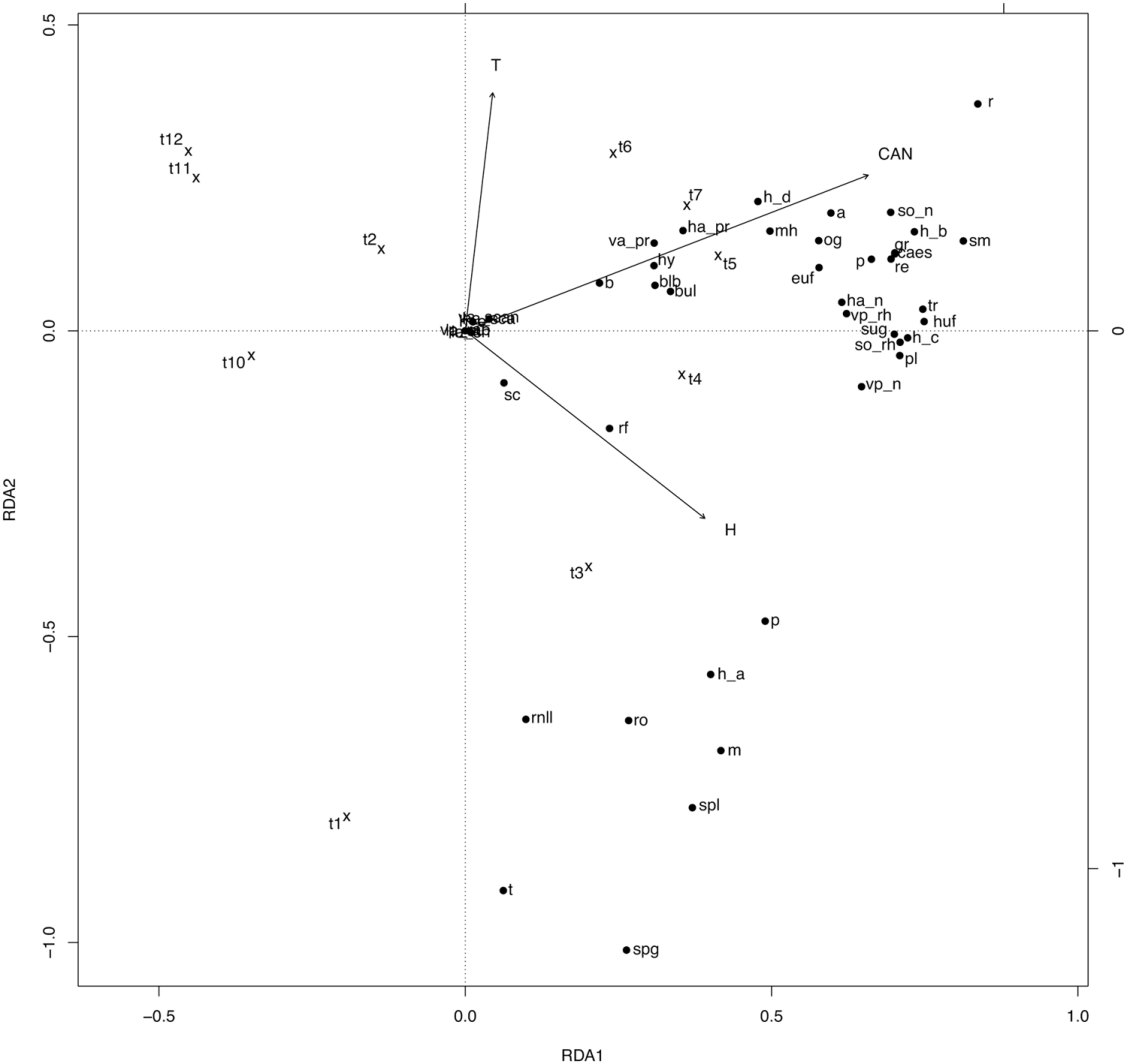
Canonical redundancy analysis ordination graph for the flat land area data set, performed on the matrix “plots-by-*F*_*x,t*_ (mean proportion of flowering shoots occurring in plot *x* at time *t*, for species sharing a trait state)”, constrained by soil temperature (T), soil relative humidity (H), canopy height (CAN) and observation time (t1–4 Apr; t2–18 Apr; t3–2 May; t4–16 May; t5–30 May; t6–13 Jun; t7–30 Jun; t8–20 Jul; t9–5 Aug; t10–19 Aug; t11–6 Sep; t12–21 Oct). The legend is the same as that of Fig. 1.

The RDA ordination graphs (Figs 1–3) showed a general partition of trait states into three main groups for south and north facing slopes (related to high soil humidity, low temperature and canopy height values; low soil humidity, high soil temperature and canopy height values, and intermediate values of the environmental variables), and two main groups in flat lands (related to high soil humidity and low temperature, and to high canopy height values, before hay mowing).

Linear mixed-effects models (Table 2) indicated that most of the trait-related flowering trends are context-dependent, i.e. related to the topographic location of the grassland community. Indeed, in the south-facing community they were more frequently related to temperature and canopy height, besides time (e.g. annual life form, plant height ≤20 cm, persistent tap root, spring and summer green leaves, mesomorphic and succulent/hygromorphic leaves, reptant and rosette growth form). In the north-facing and in the flat land community, models indicated the significant influence of a higher number of environmental variables together, in comparison with south-facing slopes (e.g. absence of vegetative propagation, bulbils, persistent tap root, spring green and summer green leaves, scleromorphic-mesomorphic leaves and reptant growth form).

**Table 2:** Models explaining the effect of soil temperature, soil relative humidity, canopy height and time of observation, on mean flowering proportion of trait states, in the whole study area (W) and in the grassland communities located on north-facing slopes (N), south-facing slopes (S), and flat lands (F).

Trait states	Dataset	Model	BIC full model	BIC null model	logLik full model	logLik null model	*P*
*Life span*							
Annual	W	top + T + H + can + time	906.6	1078.7	−389.5	−496.8	***
	S	can + time	416.4	464.9	−178.0	−214.3	***
	N	T + H + can + time	393.5	418.9	−166.6	−191.3	***
	F	T + H + can	−32.7	169.7	45.6	−67.3	***
Biennial	W	top + can + time	207.1	281.4	−39.8	−98.2	***
	S	can + time	185.5	186.6	−62.6	−75.2	***
	N	H + can	58.4	54.9	1.0	−9.3	***
	F	T + H + can	−51.2	41.8	54.9	−3.3	***
Perennial	W	top + T + can + time	−65.7	399.6	96.6	−157.3	***
	S	T + time	−65.1	83.2	62.7	−23.5	***
	N	T + H + can + time	−152.3	189.1	106.3	−76.4	***
	F	T + time	11.1	109.9	23.8	−37.4	***
*Vegetative propagation*							
Absent	W	top + T + can + time	554.5	866.8	−213.51	−390.9	***
	S	T + can + time	14.8	129.8	22.80	−46.8	***
	N	T + H + can + time	243.5	421.9	−91.5	−192.8	***
	F	T + H + can + time	126.2	233.5	−33.8	−99.2	***
Root, tuber, bulb splitter	W	T + H + time	1064.4	1212.7	−468.5	−563.9	***
	S	H + can + time	350.0	398.5	−144.8	−181.1	***
	N	T + H + time	444.1	499.2	−191.9	−231.5	***
	F	T + H + can + time	228.9	299.7	−85.2	−132.3	***
Bulbils	W	top + can + time	272.2	359.6	−72.4	−137.3	***
	S	H	34.8	39.2	12.8	−1.5	***
	N	H + time	161.9	156.2	−50.8	−60.0	**
	F	T + H + can + time	55.9	167.2	1.3	−66.1	***
Runner, runner−like rhizome	W	top + T + H + can + time	1044.7	1338.3	−458.6	−626.7	***
	S	T + H + can + time	376.7	452.1	−158.2	−207.9	***
	N	T + can + time	397.5	486.8	−168.6	−225.3	***
	F	T + H + can	223.2	412.5	−82.3	−188.7	***
Rhizome	W	top + T + H + can + time	670.5	954.3	−271.5	−434.7	***
	S	T + H + can + time	370.4	465.2	−155.0	−214.5	***
	N	T + H + can + time	196.8	324.2	−68.2	−144.0	***
	F	can + time	−27.3	111.4	42.9	−38.1	***
*Storage organ*							
Absent	W	top + T + H + can + time	1027.8	1247.7	−450.2	−581.4	***
	S	H + can + time	391.0	468.3	−165.3	−216.0	***
	N	T + can + time	441.1	514.6	−190.4	−239.2	***
	F	T + H + can	99.3	250.4	−20.3	−107.6	***
Rhizome	W	top + T + H + can + time	551.2	928.9	−211.85	−422.0	***
	S	T + H + can + time	310.9	458.1	−125.2	−210.9	***
	N	T + H + can + time	62.1	270.0	−0.8	−116.9	***
	F	T + can + time	40.7	159.0	8.9	−61.9	***
Bulb	W	top + can + time	724.2	831.6	−298.4	−373.3	***
	S	H + can + time	270.4	306.2	−105.0	−135.0	***
	N	T + H + can + time	323.9	356.4	−131.7	−160.1	***
	F	T + H + can + time	63.2	167.2	−2.3	−66.1	***
Tuber	W	top + T + H + can + time	1062.4	1197.3	−467.45	−556.15	***
	S	T + can + time	310.7	338.0	−125.14	−150.86	***
	N	T + H + time	344.45	388.6	−142.0	−176.2	***
	F	T + can + time	374.9	457.0	−158.1	−210.9	***
Persistent tap root	W	top + T + H + can + time	457.9	855.9	−165.2	−385.5	***
	S	T + time	−79.1	125.6	69.8	−44.7	***
	N	T + H + can + time	174.1	411.5	−56.8	−187.6	***
	F	T + H + can + time	64.3	240.6	−2.9	−102.7	***
*Leaf persistence*							
Persistent green	W	top + T + H + can + time	250.1	727.8	−61.3	−321.4	***
	S	T + H + can + time	64.2	264.4	−1.9	−114.1	***
	N	T + H + can + time	130.5	360.6	−35.1	−162.2	***
	F	T + H + can + time	−138.1	68.5	98.3	−16.7	***
Summer green	W	top + T + can + time	749.6	1100.0	−311.1	−507.51	***
	S	T + time	271.2	368.6	−105.4	−166.2	***
	N	T + H + can + time	139.2	340.6	−39.4	−152.2	***
	F	T + H + can + time	296.2	388.8	−118.8	−176.8	***
Spring green	W	top + H + can + time	674.2	868.4	−273.4	−391.7	***
	S	can + time	234.0	263.4	−86.8	−113.6	***
	N	T + H + time	215.8	270.2	−77.7	−117.0	***
	F	H + can + time	171.0	315.6	−56.2	−140.2	***
Overwintering green	W	top + H + can + time	597.5	784.3	−235.0	−349.7	***
	S	T + H + can + time	313.8	388.3	−126.7	−176.1	***
	N	time	191.7	198.0	−65.7	−80.9	***
	F	T + H + can + time	−2.1	171.9	30.3	−68.4	***
*Leaf anatomy*							
Succulent	W	top + T + time	−770.2	−726.8	448.8	405.9	***
	S	H + can	129.3	160.1	−34.5	−61.9	***
	N		−1000.0	−1019.9	530.1	528.1	n.s.
	F	—	—	—	—	—	—
Succulent/ hygromorphic	W	top + T + time	−313.1	−268.2	220.3	176.6	***
	S	time	40.0	57.9	10.2	−10.8	***
	N	H + time	15.5	31.9	22.5	2.2	***
	F		−1001.4	−1024.4	530.0	529.8	n.s.
Hygromorphic	W	top + T + H + can + time	−1294.1	−830.0	710.8	457.5	***
	S	—	—	—	—	—	—
	N		−712.3	−730.6	386.4	383.4	n.s.
	F	T + H + can + time	−119.1	144.3	88.9	−54.6	***
Mesomorphic	W	top + T + can + time	685.7	972.0	−279.1	−443.5	***
	S	T + time	350.2	419.6	−144.9	−191.7	***
	N	T + can + time	121.9	219.2	−30.7	−82.4	***
	F	T + time	147.5	217.3	−44.4	−91.1	***
Mesomorphic/hygromorphic	W	topH + can + time	351.9	652.5	−112.2	−283.8	***
	S	T + H + time	18.0	97.0	21.2	−30.4	***
	N	H + time	285.6	307.4	−112.6	−135.6	***
	F	T + H + can	11.4	230.0	23.6	−97.4	***
Scleromorphic	W	top + T + time	851.6	1009.7	−362.1	−462.4	***
	S	T + can + time	168.0	307.1	−53.8	−135.4	***
	N	T + H + time	435.4	530.2	−187.5	−247.0	***
	F	T + can + time	82.6	77.1	−12.0	−21.0	**
Scleromorphic/mesomorphic	W	top + T + H + can + time	798.2	1102.8	−335.4	−509.0	***
	S	can + time	253.8	357.3	−96.7	−160.5	***
	N	T + H + can + time	112.9	352.6	−26.2	−158.2	***
	F	T + H + can + time	258.4	378.6	−99.9	−171.7	***
*Horizontal space occupation*							
Absent	W	top + T + H + can + time	788.4	1132.6	−330.5	−523.8	***
	S	T + can + time	253.9	403.0	−96.7	−183.4	***
	N	T + H + can + time	360.1	492.0	−149.9	−227.9	***
	F	T + H + can + time	44.1	211.6	7.2	−88.2	***
Caespitose	W	top + T + H + can + time	683.5	879.7	−278.0	−397.4	***
	S	T + H + can + time	308.7	406.2	−124.1	−185.0	***
	N	T + can + time	197.4	293.0	−68.5	−128.4	***
	F	T + H + can + time	51.5	179.9	3.5	−72.4	***
Pleiocorm	W	top + T + H + can + time	467.1	955.7	−169.8	−435.4	***
	S	T + H + can + time	152.9	314.4	−46.3	−139.1	***
	N	T + H + can + time	38.2	380.8	11.103	−172.3	***
	F	T + H + can + time	129.4	268.2	−35.4	−116.5	***
Reptant	W	top + T + H + can + time	1127.8	1314.2	−500.2	−614.6	***
	S	can + time	368.6	451.3	−154.1	−207.5	***
	N	T + H + can + time	302.6	422.7	−121.1	−193.3	***
	F	T + can + time	416.5	446.3	−179.0	−205.6	***
Rosulate	W	top + T + time	924.5	1112.7	−398.52	−513.9	***
	S	T + time	324.4	405.2	−132.0	−184.5	***
	N	T + H + time	332.6	401.6	−136.1	−182.7	***
	F	H + time	258.2	309.5	−99.8	−137.2	***
Climber	W	can	−3088.1	−3078.3	1607.8	1581.6	***
	S	—	—				—
	N		−1288.0	−1310.2	674.2	673.2	n.s.
	F	can	−537.5	−540.7	298.0	287.9	***
Prostrate	W	top + can + time	−508.3	−434.7	317.9	259.8	***
	S	can	−236.4	−249.3	148.4	142.8	*
	N	time	−1071.0	−1091.3	565.7	563.8	n.s.
	F	can + time	133.6	173.0	−37.5	−68.9	***
*Vertical space occupation*							
No leafy stem with narrow basal leaves	W	top + T + can + time	252.9	358.0	−62.7	−136.5	***
	S	T + H + time	9.0	81.9	25.7	−22.8	***
	N	T + H	−276.6	−278.7	168.5	157.5	***
	F	T + H + can + time	233.9	330.9	−87.7	−147.8	***
Grass	W	top + T + H + can + time	658.9	953.5	−265.7	−434.3	***
	S	T + H + can + time	319.8	381.5	−129.7	−172.7	***
	N	T + can + time	250.4	403.5	−95.0	−183.6	***
	F	T + H + can + time	10.0	158.2	24.3	−61.5	***
Rosette forbs	W	top + time	997.8	1122.5	−435.2	−518.8	***
	S	T + time	343.4	424.1	−141.50	−193.9	***
	N	T + H + can + time	349.4	411.9	−144.47	−187.8	***
	F	T + time	255.0	261.8	−98.22	−113.3	***
Hemirosulate upright forb	W	top + T + H + can + time	545.8	958.4	−209.17	−436.7	***
	S	T + H + can + time	236.6	384.2	−88.10	−174.0	***
	N	T + H + can + time	82.7	325.5	−11.17	−144.6	***
	F	T + H + can + time	73.3	88.3	−7.374	−17.8	***
Erosulate upright forb	W	top + T + H + can + time	804.9	1160.9	−338.7	−538.0	***
	S	T + H + can + time	275.0	428.4	−107.3	−196.1	***
	N	T + H + can + time	348.2	389.3	−143.9	−167.5	***
	F	T + H + can + time	148.7	257.7	−45.0	−111.3	***
Climber	W	can	−3088.1	−3078.3	1607.8	1581.6	***
	S	—	—	—	—	—	—
	N		−1288.0	−1310.2	674.2	673.2	n.s.
	F	can	−537.5	−540.7	298.0	287.9	***
Prostrate forb	W	top + T + H + can + time	336.5	417.1	−104.5	−166.1	***
	S	T + H + can	86.1	148.189	−12.8	−56.0	***
	N	T + H + can + time	108.7	158.40	−24.14	−61.1	***
	F	T + can + time	112.3	131.14	−26.865	−48.0	***
*Plant height (cm)*							
≤20	W	top + T + can + time	397.0	686.3	−134.8	−300.7	***
	S	T + time	−11,3	113.0	35.8	−38.4	***
	N	T + H + time	282.6	386.1	−111.1	−175.0	***
	F	T + time	59.4	126.3	−0.4	−45.6	***
21−40	W	top + T + H + can + time	836.0	1159.3	−354.3	−537.2	***
	S	T + H + can + time	253.8	423.5	−96.7	−193.6	***
	N	T + H + can + time	400.1	503.8	−169.9	−233.8	***
	F	T + H + can + time	31.0	32.2	13.8	10.2	***
41−60	W	top + T + can + time	844.3	1110.3	−358.4	−512.7	***
	S	T + can + time	398.6	420.4	−169.1	−192.1	***
	N	T + can + time	207.1	394.2	−73.3	−179.0	***
	F	can + time	189.9	302.0	−65.7	−133.4	***
61−80	W	top + H + can	−794.2	−481.2	460.8	283.1	***
	S	—	—	—	—	—	—
	N		−479.0	−500.7	269.7	268.5	n.s.
	F	T + H + can	101.4	231.4	−21.4	−98.1	***
81−100	W	can	−3647.8	−3675.8	1887.6	1880.4	*
	S	—	—	—	—	—	—
	N	can	−1305.5	−1322.1	683.0	679.2	n.s.
	F	H	−766.3	−781.28	412.4	408.2	n.s

Models were performed using linear mixed-effects modelling through *lm4* R package (lmer function). Statistical significance (*P*) of differences between the full model and the null model (with and without all the fixed effects, respectively) was determined by likelihood ratio tests. BIC and logLik values for the full models and the null models are shown. The model formulas included the variables that contributed significantly to the models (*P* < 0.05).

top - topographic position, T - soil temperature, H - soil relative humidity, can - canopy height, *P* - level of statistical significance of differences from the full model and the null model. ^***^ *P* < 0.001, ^**^ *P* < 0.01, ^*^ *P* < 0.05.

The species composition of the three studied plant communities is shown in the Supporting information Data set, highlighting that *Bromus erectus* and *Helianthemum oelandicum* subsp. *incanum* are the dominant species (i.e. the most abundant species, with mean cover greater than 25%) on south-facing slopes, while *B. erectus* and *Festuca rubra* s.l. on north-facing slopes, and *B. erectus*, *Cynosurus cristatus*, *Lolium perenne*, *Poa trivialis* and *Trifolium pratense* on flat lands.

## Discussion

Our results indicated that in early spring (observation times 1–3/4, with high soil humidity, low soil temperature and canopy height), flowering species in each plant community tend to be equipped with trait attributes (overwintering and spring green leaves, bulbs, tubers and small size) devoted to a fast growth and blooming strategy^25,26^, likely related to the need to predate the tallest and dominant species and to face competition for light and other resources^27,28^. Moreover, some of these traits (mesomorphic or mesomorphic/hygromorphic leaves) may be considered a by-product of the high soil humidity, also because most of the early-flowering species (with overwintering and spring green leaves) are ending the reproductive cycle before the onset of summer. Instead, species blooming under optimal conditions (i.e. low or absent water stress, intermediate temperature and high light availability, fostering the high available N amount in the soil^29^ and a high photosynthetic rate - observation times 4–7) exhibited two sets of strategies, one (i.e. rhizomes, grass and caespitose growth form, persistent green leaves^10,30^) shown by the dominant species (as indicated in the Supporting information Data sets), and another used by the subordinate ones, devoted to minimizing water loss by evapotranspiration (erosulate upright forbs, scleromorphic and scleromorphic/mesomorphic leaves) or aimed at maximizing the species’ competitive ability allowing individuals to explore the neighboring areas (runner/runner-like rhizomes, reptant growth form^31^).

Our results highlighted a quite general pattern among the three plant communities based on a change in strategies exhibited by species in bloom as the season progresses^11^. In the early flowering phases, there is a predominance of species with strategies of slow resource acquisition and storage but rapid growth rate, while in the periods with optimal environmental conditions, the blooming species are the ones with strategies of slow growth rate and more efficient resource acquisition, conservation and use. Instead, no common patterns emerged among plant communities for the late flowering species from mid-summer to autumn.

We observed also that the temporal shift in soil temperature, soil relative humidity and canopy height among communities (see Supplementary Fig. S2) was reflected in a temporal modification of the flowering expression of some traits, such as leaf anatomy and persistence, type of storage organ and vertical space occupation (Supplementary Table S1). These seeming differences further support the relationship of some traits with the occurrence of specific environmental conditions, regardless of the type and productivity of plant community.

Instead, variation partitioning analysis indicated that the greater the productivity, the higher the variance explained by variation of canopy height, soil temperature and soil relative humidity. At the more productive extreme of the gradient, we observed the highest number of indicator trait states reflecting strategies devoted to the storage of resources and competition for light (type of storage organs, leaf persistence, plant height, horizontal and vertical space occupation type) in the phases of the growing season (observation times 4–7), when the community is reaching its peak of canopy height (Supplementary Fig. S2c, Supplementary Table S1). Conversely, in the plant community of south-facing slopes, which are characterized by low soil moisture in early spring and humidity values close to zero during the summer (Supplementary Fig. S2a), likely reflecting in very low nutrient availability^32^, strategies allow both drought resistance and avoidance ability, such as small size, bulbs, succulent/hygromorphic leaves and rosulate habit^33,34^ (Table 1, Supplementary Table S1 and Fig. 1). Moreover, late flowering species were equipped with trait attributes (succulent leaves, prostrate growth form, annual and biennial life histories) that are typical of plants adapted to dry habitats^27,35,36^. The observation of species with short life span lends support to Sun & Frelich’s assertions^12^ that late flowering species may exhibit low investment strategies, as indicated by our results in dry environments.

In general, our results seem to substantiate the hypothesis that temporal variation of resources plays a role in trait differentiation and richness within a plant community and that the functional response to the seasonal variation in environmental conditions largely retraces the modifications, at the community level, of the functional composition across spatial resource gradients^14,15,36^. In fact, our results are consistent with findings suggesting that harsh conditions strongly filter species with resource-retaining strategies^26^, drought resistance and avoidance ability^33,34^ and that in productive conditions, competition for light is a paramount driver in filtering trait composition of grasslands, promoting the coexistence of species with dissimilar resource acquisition strategies on a fine spatial scale^37^.

Moreover, our findings seem to be consistent with the fluctuation niche theory^20^, based on the different growth/phenological response of species to the temporal variation of resource availability. New insight into the effect of niche fluctuation was provided by variation partitioning and general linear modelling results (Table 2), which indicated that in productive conditions (having the highest differences in soil relative humidity and canopy height between spring and summer), the trait-related flowering pattern was more influenced by environmental fluctuations in time than it was in less productive conditions. We found that in productive conditions, the phenological responses to environmental fluctuations are mostly related to traits that limit competition with dominant species by spatial niche segregation (vegetative propagation, vertical space occupation, and plant height) and to species that fit their life cycle to the variation of environmental conditions, through different life and leaf span. Conversely, such traits showed weaker trends in the driest community, where the flowering pattern was less dependent on temporal fluctuations of environmental conditions, confirming that drought stress and the likely related shortage of soil nutrients act as strong drivers in filtering the trait composition of plant communities. In addition, results of linear mixed-effects modelling (Table 2) indicated that in dry habitats, traits related to strategies of drought stress tolerance or avoidance, e.g. annual life form, small size, spring green leaves, persistent tap root, reptant, prostrate and rosulate growth forms^10,26,35,38^, responded to the fluctuations of single environmental variables. It also emerged that the temporal gradient, which synthesizes the variation trend of the environmental conditions and vegetation structure of the grassland communities during the growing season, is a major factor in determining the trait-based flowering pattern in grassland communities. Topographic factors, which condense a set of environmental conditions that result in different available water capacities of soils^23^, emerged as a key element as well.

Finally, some differences in the trait-related flowering patterns could be due to the different management types. In fact, in mowed meadows, species equipped with tubers and rosettes were in bloom in the late phases of the growing season after hay removal (Supplementary Table S1). Re-sprouting from tubers is considered a tolerance strategy that follows a non-selective disturbance, while the rosette growth form is an escape strategy allowing plants to survive disturbance and exploit the newly available spatial niches, taking advantage of very low canopy height and low competition for light^27^.

## Conclusion

Our results revealed that in the study case the trait-related response of flowering phenology follows complex patterns, driven by the interplay of the context-dependent environmental conditions and the temporal fluctuation of resources. In fact, we observed that the mechanism behind the flowering pattern follows a general model regardless of the productivity level (since many traits are strongly linked to the temporal variation of environmental conditions), but that the amplitude of the environmental fluctuations influences the number of strategies positively filtered by the system, being the more productive condition related to a wider range of environmental fluctuation. As far as we know, our research gives a first experimental validation of the effectiveness of the fluctuation niche theory, based on a trait-based phenological study.

## Methods

### Study area

The study area is located on the calcareous ridge of central Italy (42°56′53″N; 13°00′35″E), between 1000 and 1100 m a.s.l. It is characterized by alternation of winter cold stress and summer drought stress with a mean annual temperature of 10 °C, annual rainfall of 1035 mm, and a drought period occurring from mid-July to the end of August; the average growing season lasts from late April to October^39^. Two of the three studied perennial grassland communities are dominated by *Bromus erectus* and were traditionally devoted to grazing, while one, dominated by *Cynosurus cristatus*, was dedicated to hay production. The first grazed community, located on south-facing slopes, is characterized by xeric pastures with open sward, growing on shallow rocky soils (5–15 cm of depth). The second grazed community, located on north-facing slopes, has a dense sward and grows on soils of 30–50 cm. The hay meadows grow on flat areas (bottom of the valley), with deep soils (80–120 cm)^23^. The average aboveground dry phytomass ranges from 70 to 100 g m^−2^ yr^−1^ on south-facing slopes, 150 to 200 g m^−2^ yr^−1^ on north-facing slopes, and 250 to 300 g m^−2^ yr^−1^ on flat areas^40^. Apart from the abovementioned differences (reflecting in different potential available water content)^23^, such soils have similar pH (6.0–7.0), nitrogen content (6.5–7.0 g/Kg) and texture (clay: 50–60%; silt 30–40%; sand 8–10%)^23^. This is because they originated from former soils that were differentially eroded (because of the different slope angle and aspect), giving origin to a “soil chain” characterized by different soil depth^23^. Such soil differences mostly reflect in community species composition, being the xeric community mostly composed of chamephytes and small-sized species with avoidance strategies, while the others are composed of tall hemicroptophytes, with tolerance strategies, which originate stratified layers. At present, the xeric and semi-mesophilous pastures on the slopes are grazed by sheep and cows, with a quite homogeneous real livestock pressure (1.0–1.2 livestock units per hectare), while the hay meadows are mown once a year (in July) and then grazed by cows.

### Sampling design and data collection

Data were collected, in fenced 1 m × 1 m plots_,_ twelve times during the 2011 growing season (4 Apr, 18 Apr, 2 May, 16 May, 30 May, 13 Jun, 30 Jun, 20 Jul, 5 Aug, 19 Aug, 6 Sep, and 21 Oct). In each plot, we counted the number of flowering shoots in full bloom per species at each time. We defined ‘in full bloom’ as the flowering of more than 70% of the buds, since we considered this threshold value a good indicator to define the moment when individuals are at their utmost reproductive effort. On meadows community, sampling was not conducted on 20 Jul and 5 Aug because these were times when hay was mown to maintain the traditional marked change in community height, lowering the turf height to 2–3 cm. Instead, grazing was not allowed to avoid the cows trampling and herbage removal and because, as sheep actively select flowers^41^, this would have caused a not accurate count of the number of flowering shoots. Based on previous studies^10,13^, we assumed that the time interval between two consecutive observations was wide enough to record the flowering time for all the species and to avoid counting flowering shoots twice during the subsequent times of sampling. We chose to conduct the field work in one year to avoid problems related to inter-annual climate variations, likely leading to shifts in the phenological events and, hence, making more difficult the comparison between the two years (in relation to the aim of our study). Moreover, inter-annual rainfall variation (normal condition in the sub-Mediterranean climate) could modify the relative abundance of species and the number of flowering shoots.

To reduce the environmental variability among the observational units, we selected an area, homogeneous in terms of altitude (1000–1100 m a.s.l.), geological substrate (limestone) and main soil features (i.e. pH, texture, soil nitrogen, etc.). Moreover, Blasi *et al*.^42^ ascribed the whole study area to one specific Italian biogeographic element (Temperate Division, Apennine Province, central and southern Apennine section and Umbria and Marche Apennine subsection). Using a GIS generator of random points (Q-GIS software), we randomly selected 105 points which were the left-hand corners of as many 1 m × 1 m plots (35 on north-facing slopes; 35 on south-facing slopes; 35 on flat areas). We verified that every plot of each condition had undergone traditional management during the last thirty years (sheep grazing on slopes, mowing on flat areas).

For the year 2011, we gathered meteorological data (daily temperature and daily rainfall amount) from the climatic station of the Torricchio Mountain Natural Reserve, which is located in the middle of the study area. We also recorded soil temperature (°C), soil relative humidity (%) and canopy height (cm) in each plot at each date. Soil temperature and relative humidity were measured at 10 cm of depth, using a digital field soil temperature and humidity meter (T.R. - Turoni S.r.L. 46908); data were randomly collected between 11:00 a.m. and 1:00 p.m. on cloudless days, with ten sampling replicates. Canopy height (the shortest distance between the upper boundary of the main photosynthetic tissue on a plant and the ground level) was considered as an indicator of productivity and intensity of competition for light and of the plants’ resource investment strategies, since it is associated with the ability to tolerate or avoid environmental stresses^43^. It was measured in five points randomly chosen in each plot.

The traits considered for each plant species were chosen according to indications of Lavorel *et al*.^15^, Dìaz *et al*.^44^ and Grime^27^, following the assumption that analysis of traits related to competitive ability, resource acquisition and stress tolerance may help to understand the species coexistence mechanisms. Life span, plant height, occurrence and type of vegetative propagation, type of space occupation (horizontal and vertical) and leaf persistence were selected as indicators of resource acquisition strategies. Instead, leaf anatomy, leaf persistence, and type of storage organs were selected as indicators of stress tolerance. As it was not possible to collect data on plant height in the field for all the individuals, and thus to account for the intra-specific variability within and between plots, this trait was considered as a categorical variable^14,45^. Supplementary Table S2 shows the list of traits and trait states, traits description and their respective data sources.

In order to get an overview of the species composition of the studied vegetation, we carried out 10 relevés for each community. In each 1 m × 1 m plot, we visually estimated the species cover values (%).

All data generated or analysed during this study are included in this published article and its Supplementary Information files.

### Data analysis

We calculated the average monthly temperature and precipitation values in the year 2011 and the averages for the soil relative humidity, soil temperature, and canopy height values in each plot at each observation time for each community. Subsequently, for each flowering species, we calculated for each plot the proportion of flowering shoots occurring at each time out of the total number of flowering shoots in the whole growing season (April-October). Next, we grouped species sharing the same trait state and averaged their percentage of flowering shoots, using the formula^10^:

1 $${F}_{x,t}=\frac{{\sum }_{i=1}^{S}({n}_{x,t,i}/{n}_{tot,i})}{{S}_{x,t}}$$

where *F*_*x,t*_ is the mean proportion of flowering shoots occurring in plot *x* at time *t*, for species sharing a trait state; *n*_*x,t,i*_ is the number of flowering shoots of a species counted in plot *x*, at time *t*; *n*_*tot,i*_ is the total number of flowering shoots of a species counted over the whole blooming period in plot *x*; *S*_*x,t*_ is the number of flowering species sharing the same trait state in plot *x*, at time *t*. *F*_*x,t*_ values were the elements of a “relevés-by-trait states” matrix used for the subsequent statistical processing.

To detect possible trait patterns related to the different flowering succession in the three plant communities, we used Indicator species analysis (ISA), a method that serves to discern those items that show preferential distribution in a group of samples in comparison with the other groups^46^. According to Dufrêne and Legendre^46^, ISA involves the calculation of an indicator value (IV), which is the product of relative abundance and relative frequency, using the formula

2 $$I{V}_{tj}=R{A}_{tj}\times R{F}_{tj}$$

where *RA* is the relative abundance and *RF* is the relative frequency for each trait state *j* at each time *t*. In our study, *RA* was calculated using the formula

3 $$R{A}_{t,j}=\frac{{\sum }_{x=1}^{{n}_{t}}({F}_{x,t,j})/{n}_{t}}{{\sum }_{t=1}^{g}({\sum }_{x=1}^{{n}_{t}}({F}_{x,t,j})/{n}_{t})}$$

where *F*_*x,t,j*_ is the flowering proportion of trait *j* in plot *x* of time *t*, *n*_*t*_ is the total number of sampling units at each time, and *g* is the total number of times. In other words, *RA* is the relative flowering proportion, namely, the mean flowering proportion of a trait state within an observation time divided by the sum of mean flowering proportions in all the observation times. *RF* was calculated using the formula

4 $$R{F}_{t,j}=\frac{{\sum }_{x=1}^{{n}_{t}}\,{f}_{x,t,j}}{{n}_{t}}$$

where ${\sum }_{x=1}^{{n}_{t}}\,{f}_{x,t,j}$ is the number of sampling units *x* at the observation time *t*, occupied by flowering species with the trait state *j*, and *n*_*t*_ is the total number of sampling units at each time. Once the IV is calculated, the group in which IV is at its maximum is identified^47^. IV may range from 0 (absence of flowering for all the species sharing a trait in all plots laid in a community at time *t*) to 1 (all species sharing a trait in a community are in bloom at a time *t* in all plots).

To identify the indicator trait states of every observation time in each studied plant community, we performed three ISAs (one per plant community) on the matrix “relevés-by-trait states’ *F*_*x,t*_ values”, where relevés were grouped on the basis of the observation time. We looked for indicator trait states of individual observation times and combinations of two and three observation times^48^. We tested the statistical significance (*P* < 0.05) of the observed maximum IVs using permutation tests with 4999 runs, and discarded trait states whose component of relative abundance was lower than 0.6 or whose component of relative frequency was lower than 0.25^48^.

To perform ISA we used R software (version 3.0.2 - R Foundation for Statistical Computing, Vienna, Austria http://www.R-project.org) and the R-packages *indicspecies* version 1.7.6 (*multipatt* function) and *permute* 0.9–4 (*how* function).

To assess the relation between the trait-related flowering pattern (mean proportion of flowering shoots for species sharing each trait state over time) and the seasonal variation of the environmental variables in the three plant communities, we performed for each community a canonical redundancy analysis (RDA) on the “relevé-by-trait states (*F*_*x,t*_ values)” matrix, constrained by soil temperature, soil relative humidity and canopy height (quantitative variables) and by observation time (factor with 12 classes, ten for flat areas). To assess the significance of the RDA models, we used a permutation test with 999 permutations^49^.

To discriminate the effects on flowering events (in terms of proportion of variance explained) exclusively due to the spatial environmental heterogeneity inside each community, to the temporal pattern of trait-based flowering expression, and to the temporal trend of environmental variables, the total variance was partitioned into fractions explained by each set of the predictor variables, singly and in combination, by partial RDAs; adjusted R-squared values (*adj. R* ^2^) were calculated to produce unbiased estimates of the contributions of the independent variables to the explanation of the response variables^50^. To test whether each independent fraction exhibited a significant influence on flowering data, we applied a permutation test with 999 permutations^49^. To perform RDA, variation partitioning and tests of RDA models, we used *vegan* R-package, version 2.3–4 (rda, varpart, and anova.cca functions).

To assess and compare in the three plant communities the influence of soil temperature, soil relative humidity, canopy height, and time on the trait-related flowering trends, and to evaluate the effects of the above-mentioned variables and of topographic location on the whole data set, we used linear mixed-effects modelling, fitting a model for each trait state, on the whole data set and three models on the subsets composed of plots laid in the respective topographic position.

As fixed effects we entered soil temperature, soil relative humidity, canopy height, time (quantitative variables), and topographic position (categorical variable). As random effects, we had random intercepts for topographic position and plots ID, as well as by-plot ID random slopes for the effects of time and topographic position. Fixed and random effects including topographic position were entered in the models fitted on the whole data set. To perform the elaborations, we used the *lme4* R package, version 1.1–13 (lmer function).

Significance (*P*) values were obtained by likelihood ratio tests for the full models with the fixed effects in question against the models without the effects in question. We used this type of test also to delete the variables that did not contribute significantly to the models, testing if models without a fixed effect were significantly different from those with that effect. Statistically significant difference leads to retain the variable in the model. Comparison between models was performed using the anova R function (*stats* R package, version 3.3.2).

## Electronic supplementary material

Supporting information

Supporting information data set
