# Supplementary material for: Filtering effect of temporal niche fluctuation and amplitude of environmental variations on the trait-related flowering patterns: lesson from sub-Mediterranean grasslands
Source: Sci Rep. 2017 Sep 20;7:12034. doi: 10.1038/s41598-017-12226-5 (PMC5607319; doi:10.1038/s41598-017-12226-5)

Supplementary material

Filtering effect of temporal niche fluctuation and amplitude of environmental variations on the trait-related flowering patterns: lesson from sub-Mediterranean grasslands

Andrea Catorci[1], Karina Piermarteri[2], Károly Penszka[3], Judit Házi[3] & Federico Maria Tardella[1,*]

***corresponding author (e-mail: dtfederico.tardella@unicam.it)

[1]School of Biosciences and Veterinary Medicine, University of Camerino, Via Pontoni 5, 62032 Camerino, Italy
[2]School of Advanced Studies, University of Camerino, Via Lili 55, 62032 Camerino, Italy
[3]Szent István University, Faculty of Agricultural and Environmental Sciences, Institute of Botany and Ecophysiology, 2100 Gödöllő Páter K. st.1., Hungary

Table S1: Descriptive statistics of $F_{x,t}$ values (i.e. mean proportions of flowering shoots occurring in plot x at time t, for species sharing a trait state) for each plant community at each observation time (1 - 4 Apr; 2 - 18 Apr; 3 - 2 May; 4 - 16 May; 5 - 30 May; 6 - 13 Jun; 7 - 30 Jun; 8 - 20 Jul; 9 - 5 Aug; 10 - 19 Aug; 11 - 6 Sep; 12 - 21 Oct).
F: flat land; Med.: median; N: north-facing slope; qu.: quartile; S: south-facing slope; SD: standard deviation; Top.: topographic position of grassland communities

Observation time 1–4

Traits and trait states	Top.	1 Mean	SD	Med.	1st qu.	3rd qu.	2 Mean	SD	Med.	1st qu.	3rd qu.	3 Mean	SD	Med.	1st qu.	3rd qu.	4 Mean	SD	Med.	1st qu.	3rd qu.
Life span																					
Annual	S	0.00	0.00	0.00	0.00	0.00	0.38	0.41	0.17	0.00	0.73	0.68	0.43	0.95	0.24	1.00	0.12	0.30	0.00	0.00	0.01
	N	0.00	0.00	0.00	0.00	0.00	0.09	0.28	0.00	0.00	0.00	0.51	0.51	1.00	0.00	1.00	0.20	0.39	0.00	0.00	0.00
	F	0.00	0.00	0.00	0.00	0.00	0.11	0.30	0.00	0.00	0.00	0.20	0.39	0.00	0.00	0.00	0.37	0.30	0.34	0.12	0.58
Biennial	S	0.00	0.00	0.00	0.00	0.00	0.33	0.44	0.00	0.00	0.78	0.03	0.17	0.00	0.00	0.00	0.14	0.33	0.00	0.00	0.00
	N	0.00	0.00	0.00	0.00	0.00	0.00	0.00	0.00	0.00	0.00	0.00	0.00	0.00	0.00	0.00	0.21	0.41	0.00	0.00	0.00
	F	0.00	0.00	0.00	0.00	0.00	0.00	0.00	0.00	0.00	0.00	0.05	0.21	0.00	0.00	0.00	0.00	0.00	0.00	0.00	0.00
Perennial	S	0.97	0.17	1.00	1.00	1.00	0.59	0.22	0.58	0.48	0.71	0.73	0.12	0.72	0.66	0.79	0.62	0.14	0.64	0.57	0.72
	N	0.53	0.34	0.56	0.22	0.82	0.57	0.24	0.54	0.38	0.68	0.53	0.12	0.49	0.45	0.62	0.72	0.09	0.70	0.68	0.80
	F	0.78	0.22	0.75	0.54	1.00	0.37	0.17	0.37	0.24	0.52	0.59	0.13	0.60	0.53	0.67	0.62	0.10	0.60	0.56	0.69
Vegetative propagation																					
Absent	S	0.97	0.17	1.00	1.00	1.00	0.54	0.39	0.61	0.09	0.95	0.78	0.30	0.92	0.74	1.00	0.58	0.23	0.56	0.47	0.70
	N	0.34	0.48	0.00	0.00	1.00	0.21	0.41	0.00	0.00	0.00	0.61	0.46	1.00	0.00	1.00	0.79	0.29	0.89	0.68	1.00
	F	0.21	0.40	0.00	0.00	0.00	0.26	0.41	0.00	0.00	0.59	0.62	0.40	0.80	0.34	1.00	0.44	0.26	0.45	0.22	0.62
Root, tuber or bulb splitter	S	0.00	0.00	0.00	0.00	0.00	0.42	0.39	0.40	0.06	0.73	0.72	0.20	0.73	0.61	0.87	0.51	0.24	0.55	0.41	0.68
	N	0.09	0.28	0.00	0.00	0.00	0.09	0.27	0.00	0.00	0.00	0.50	0.44	0.53	0.00	1.00	0.78	0.28	0.86	0.71	1.00
	F	0.98	0.05	1.00	1.00	1.00	0.11	0.25	0.00	0.00	0.11	0.74	0.24	0.86	0.51	1.00	0.62	0.17	0.56	0.51	0.72
Bulbils	S	0.00	0.00	0.00	0.00	0.00	0.03	0.17	0.00	0.00	0.00	0.23	0.43	0.00	0.00	0.00	0.27	0.44	0.00	0.00	0.75
	N	0.00	0.00	0.00	0.00	0.00	0.00	0.01	0.00	0.00	0.00	0.14	0.21	0.08	0.00	0.14	0.68	0.34	0.82	0.56	0.95
	F	0.00	0.00	0.00	0.00	0.00	0.06	0.24	0.00	0.00	0.00	0.00	0.00	0.00	0.00	0.00	0.20	0.41	0.00	0.00	0.00
Runner / runner-like rhizome	S	0.00	0.00	0.00	0.00	0.00	0.23	0.43	0.00	0.00	0.00	0.12	0.29	0.00	0.00	0.01	0.61	0.35	0.67	0.31	0.95
	N	0.07	0.19	0.00	0.00	0.00	0.34	0.41	0.10	0.00	0.68	0.61	0.43	0.86	0.07	1.00	0.53	0.39	0.62	0.14	0.92
	F	0.00	0.00	0.00	0.00	0.00	0.03	0.17	0.00	0.00	0.00	0.09	0.28	0.00	0.00	0.00	0.53	0.50	1.00	0.00	1.00
Rhizome	S	0.00	0.00	0.00	0.00	0.00	0.45	0.32	0.41	0.16	0.66	0.72	0.18	0.71	0.57	0.87	0.55	0.31	0.54	0.33	0.80
	N	0.19	0.23	0.13	0.00	0.25	0.47	0.32	0.34	0.23	0.76	0.45	0.15	0.43	0.34	0.56	0.63	0.15	0.63	0.55	0.73
	F	0.04	0.07	0.00	0.00	0.05	0.33	0.19	0.36	0.17	0.50	0.46	0.22	0.49	0.32	0.63	0.53	0.19	0.57	0.41	0.63
Storage organs																					
Absent	S	0.00	0.00	0.00	0.00	0.00	0.19	0.38	0.00	0.00	0.04	0.64	0.44	0.95	0.11	1.00	0.60	0.35	0.67	0.32	0.93
	N	0.00	0.00	0.00	0.00	0.00	0.18	0.35	0.00	0.00	0.11	0.82	0.34	1.00	0.83	1.00	0.49	0.39	0.57	0.04	0.82
	F	0.00	0.00	0.00	0.00	0.00	0.11	0.30	0.00	0.00	0.00	0.21	0.39	0.00	0.00	0.17	0.59	0.43	0.68	0.04	1.00
Rhizome	S	0.00	0.00	0.00	0.00	0.00	0.47	0.30	0.48	0.17	0.68	0.73	0.15	0.72	0.67	0.84	0.63	0.17	0.66	0.51	0.77
	N	0.32	0.30	0.24	0.05	0.57	0.55	0.27	0.54	0.31	0.73	0.46	0.13	0.46	0.38	0.52	0.69	0.10	0.66	0.63	0.74
	F	0.20	0.33	0.02	0.00	0.26	0.39	0.17	0.42	0.26	0.52	0.43	0.19	0.44	0.33	0.59	0.70	0.14	0.74	0.58	0.79
Bulb	S	0.00	0.00	0.00	0.00	0.00	0.16	0.31	0.00	0.00	0.14	0.61	0.41	0.72	0.22	1.00	0.55	0.39	0.62	0.06	0.92
	N	0.03	0.17	0.00	0.00	0.00	0.03	0.17	0.00	0.00	0.00	0.22	0.30	0.12	0.00	0.23	0.69	0.34	0.82	0.56	0.95
	F	0.00	0.00	0.00	0.00	0.00	0.00	0.00	0.00	0.00	0.00	0.00	0.00	0.00	0.00	0.00	0.26	0.44	0.00	0.00	0.50
Tuber	S	0.00	0.00	0.00	0.00	0.00	0.40	0.43	0.22	0.00	0.90	0.50	0.45	0.61	0.00	1.00	0.31	0.38	0.15	0.00	0.62
	N	0.09	0.28	0.00	0.00	0.00	0.09	0.27	0.00	0.00	0.00	0.51	0.47	0.50	0.00	1.00	0.46	0.46	0.40	0.00	1.00
	F	0.98	0.05	1.00	1.00	1.00	0.11	0.25	0.00	0.00	0.11	0.93	0.15	1.00	0.94	1.00	0.03	0.12	0.00	0.00	0.00
Persistent tap root	S	0.97	0.17	1.00	1.00	1.00	0.64	0.29	0.67	0.50	0.83	0.66	0.28	0.63	0.53	0.89	0.60	0.16	0.61	0.52	0.70
	N	0.20	0.41	0.00	0.00	0.00	0.05	0.14	0.00	0.00	0.00	0.38	0.32	0.35	0.12	0.66	0.72	0.16	0.74	0.60	0.84
	F	0.00	0.00	0.00	0.00	0.00	0.12	0.31	0.00	0.00	0.00	0.67	0.38	0.80	0.49	1.00	0.47	0.18	0.50	0.39	0.58
Leaf persistence																					
Persistent green	S	0.00	0.00	0.00	0.00	0.00	0.47	0.31	0.44	0.21	0.66	0.63	0.24	0.63	0.48	0.79	0.60	0.17	0.60	0.49	0.73
	N	0.07	0.19	0.00	0.00	0.00	0.37	0.38	0.26	0.00	0.60	0.51	0.18	0.53	0.40	0.64	0.64	0.11	0.64	0.56	0.72
	F	0.04	0.07	0.00	0.00	0.05	0.19	0.17	0.14	0.06	0.28	0.33	0.19	0.28	0.18	0.48	0.50	0.14	0.47	0.40	0.60
Summer green	S	0.97	0.17	1.00	1.00	1.00	0.48	0.37	0.52	0.08	0.71	0.72	0.22	0.78	0.63	0.89	0.56	0.28	0.63	0.37	0.77
	N	0.46	0.38	0.33	0.14	0.82	0.48	0.34	0.56	0.20	0.71	0.53	0.28	0.53	0.32	0.71	0.74	0.26	0.77	0.63	0.94
	F	0.21	0.40	0.00	0.00	0.00	0.51	0.48	0.67	0.00	1.00	0.72	0.36	0.88	0.69	1.00	0.54	0.36	0.52	0.26	0.92
Spring green	S	0.00	0.00	0.00	0.00	0.00	0.09	0.27	0.00	0.00	0.00	0.68	0.46	1.00	0.00	1.00	0.40	0.50	0.00	0.00	1.00
	N	0.09	0.28	0.00	0.00	0.00	0.03	0.17	0.00	0.00	0.00	0.37	0.32	0.25	0.13	0.57	0.84	0.16	0.88	0.78	0.99
	F	0.98	0.05	1.00	1.00	1.00	0.11	0.25	0.00	0.00	0.11	0.75	0.23	0.86	0.51	1.00	0.90	0.11	0.94	0.87	0.99
Overwintering green	S	0.00	0.00	0.00	0.00	0.00	0.56	0.36	0.58	0.29	0.89	0.75	0.28	0.86	0.63	0.97	0.56	0.36	0.62	0.27	0.88
	N	0.00	0.00	0.00	0.00	0.00	0.09	0.27	0.00	0.00	0.00	0.59	0.49	1.00	0.00	1.00	0.28	0.44	0.00	0.00	0.75
	F	0.00	0.00	0.00	0.00	0.00	0.11	0.30	0.00	0.00	0.00	0.25	0.41	0.00	0.00	0.47	0.38	0.31	0.38	0.08	0.59
Leaf anatomy																					
Succulent	S	0.00	0.00	0.00	0.00	0.00	0.00	0.00	0.00	0.00	0.00	0.00	0.00	0.00	0.00	0.00	0.00	0.00	0.00	0.00	0.00
	N	0.00	0.00	0.00	0.00	0.00	0.00	0.00	0.00	0.00	0.00	0.00	0.00	0.00	0.00	0.00	0.00	0.00	0.00	0.00	0.00
	F	0.00	0.00	0.00	0.00	0.00	0.00	0.00	0.00	0.00	0.00	0.00	0.00	0.00	0.00	0.00	0.00	0.00	0.00	0.00	0.00
Succulent/hygromorphic	S	0.00	0.00	0.00	0.00	0.00	0.09	0.27	0.00	0.00	0.00	0.60	0.48	0.96	0.00	1.00	0.11	0.32	0.00	0.00	0.00

Observation time 5–8

Traits and trait states	Top.	5 Mean	SD	Med.	1st qu.	3rd qu.	6 Mean	SD	Med.	1st qu.	3rd qu.	7 Mean	SD	Med.	1st qu.	3rd qu.	8 Mean	SD	Med.	1st qu.	3rd qu.
Life span																					
Annual	S	0.68	0.37	0.81	0.47	1.00	0.69	0.33	0.77	0.52	1.00	0.35	0.44	0.00	0.00	0.79	0.06	0.24	0.00	0.00	0.00
	N	0.53	0.43	0.70	0.00	0.98	0.70	0.39	0.90	0.38	1.00	0.30	0.43	0.00	0.00	0.72	0.06	0.24	0.00	0.00	0.00
	F	0.57	0.16	0.57	0.43	0.71	0.51	0.14	0.51	0.42	0.59	0.39	0.17	0.38	0.26	0.52					
Biennial	S	0.26	0.37	0.00	0.00	0.46	0.31	0.40	0.00	0.00	0.65	0.02	0.07	0.00	0.00	0.00	0.00	0.00	0.00	0.00	0.00
	N	0.30	0.45	0.00	0.00	0.96	0.24	0.43	0.00	0.00	0.27	0.03	0.17	0.00	0.00	0.00	0.00	0.00	0.00	0.00	0.00
	F	0.46	0.49	0.00	0.00	1.00	0.16	0.36	0.00	0.00	0.00	0.00	0.00	0.00	0.00	0.00					
Perennial	S	0.61	0.12	0.63	0.53	0.69	0.73	0.07	0.71	0.67	0.78	0.70	0.12	0.72	0.62	0.78	0.69	0.17	0.71	0.60	0.80
	N	0.65	0.12	0.65	0.57	0.75	0.72	0.09	0.73	0.67	0.76	0.79	0.07	0.79	0.75	0.84	0.74	0.14	0.74	0.66	0.83
	F	0.52	0.09	0.52	0.49	0.59	0.45	0.09	0.46	0.40	0.48	0.66	0.07	0.65	0.59	0.72					
Vegetative propagation																					
Absent	S	0.68	0.11	0.68	0.59	0.77	0.64	0.09	0.66	0.58	0.73	0.64	0.16	0.61	0.50	0.77	0.53	0.27	0.49	0.38	0.73
	N	0.64	0.23	0.67	0.52	0.82	0.75	0.11	0.76	0.67	0.84	0.77	0.11	0.77	0.70	0.83	0.73	0.21	0.74	0.62	0.89
	F	0.58	0.12	0.58	0.51	0.66	0.49	0.12	0.45	0.42	0.56	0.43	0.12	0.41	0.34	0.55					
Root, tuber or bulb splitter	S	0.36	0.41	0.14	0.00	0.73	0.61	0.45	0.93	0.04	1.00	0.17	0.34	0.00	0.00	0.00	0.14	0.36	0.00	0.00	0.00
	N	0.60	0.37	0.70	0.20	1.00	0.72	0.37	1.00	0.55	1.00	0.37	0.44	0.00	0.00	0.85	0.05	0.21	0.00	0.00	0.00
	F	0.36	0.19	0.34	0.24	0.43	0.45	0.27	0.54	0.19	0.66	0.40	0.21	0.51	0.15	0.53					
Bulbils	S	0.14	0.36	0.00	0.00	0.00	0.04	0.19	0.00	0.00	0.00	0.00	0.00	0.00	0.00	0.00	0.00	0.00	0.00	0.00	0.00
	N	0.16	0.27	0.00	0.00	0.21	0.00	0.01	0.00	0.00	0.00	0.00	0.00	0.00	0.00	0.00	0.25	0.43	0.00	0.00	0.34
	F	0.66	0.48	1.00	0.00	1.00	0.03	0.17	0.00	0.00	0.00	0.00	0.00	0.00	0.00	0.00					
Runner / runner-like rhizome	S	0.38	0.26	0.32	0.20	0.56	0.80	0.21	0.88	0.68	0.95	0.64	0.38	0.68	0.43	1.00	0.51	0.50	0.70	0.00	1.00
	N	0.63	0.37	0.70	0.37	1.00	0.68	0.25	0.67	0.53	0.84	0.81	0.18	0.86	0.77	0.92	0.47	0.43	0.53	0.00	1.00
	F	0.45	0.32	0.44	0.15	0.65	0.76	0.27	0.91	0.60	0.99	0.96	0.08	1.00	0.95	1.00					
Rhizome	S	0.44	0.38	0.37	0.04	0.79	0.89	0.15	1.00	0.81	1.00	0.81	0.24	0.87	0.74	1.00	0.68	0.38	0.89	0.29	1.00
	N	0.66	0.14	0.68	0.56	0.75	0.68	0.20	0.67	0.54	0.82	0.71	0.29	0.75	0.61	1.00	0.30	0.44	0.00	0.00	0.88
	F	0.55	0.17	0.52	0.47	0.61	0.21	0.16	0.20	0.07	0.36	0.50	0.25	0.43	0.32	0.72					
Storage organs																					
Absent	S	0.68	0.21	0.68	0.58	0.82	0.78	0.24	0.83	0.68	0.98	0.42	0.44	0.13	0.00	0.89	0.28	0.46	0.00	0.00	0.97
	N	0.76	0.27	0.83	0.68	0.96	0.67	0.33	0.70	0.51	1.00	0.91	0.14	1.00	0.86	1.00	0.29	0.42	0.00	0.00	0.68
	F	0.48	0.17	0.53	0.36	0.57	0.49	0.13	0.50	0.41	0.60	0.58	0.12	0.58	0.48	0.65					
Rhizome	S	0.58	0.30	0.66	0.41	0.77	0.87	0.15	0.94	0.78	1.00	0.86	0.11	0.86	0.78	0.97	0.82	0.26	0.93	0.74	1.00
	N	0.63	0.12	0.62	0.55	0.72	0.64	0.20	0.62	0.52	0.79	0.78	0.17	0.80	0.72	0.88	0.56	0.34	0.58	0.23	0.88
	F	0.50	0.11	0.52	0.42	0.57	0.33	0.17	0.37	0.21	0.48	0.70	0.16	0.67	0.58	0.84					
Bulb	S	0.15	0.35	0.00	0.00	0.00	0.07	0.25	0.00	0.00	0.00	0.11	0.32	0.00	0.00	0.00	0.14	0.36	0.00	0.00	0.00
	N	0.17	0.27	0.00	0.00	0.28	0.31	0.43	0.00	0.00	0.80	0.47	0.45	0.33	0.00	1.00	0.30	0.42	0.00	0.00	0.67
	F	0.66	0.48	1.00	0.00	1.00	0.03	0.17	0.00	0.00	0.00	0.00	0.00	0.00	0.00	0.00					
Tuber	S	0.81	0.14	0.82	0.71	0.94	0.29	0.22	0.21	0.11	0.51	0.00	0.00	0.00	0.00	0.00	0.00	0.00	0.00	0.00	0.00
	N	0.58	0.43	0.67	0.09	1.00	0.58	0.44	0.77	0.00	1.00	0.00	0.00	0.00	0.00	0.00	0.00	0.00	0.00	0.00	0.00
	F	0.00	0.00	0.00	0.00	0.00	0.00	0.02	0.00	0.00	0.00	0.62	0.49	1.00	0.00	1.00					
Persistent tap root	S	0.59	0.12	0.60	0.50	0.66	0.63	0.10	0.64	0.54	0.70	0.65	0.14	0.65	0.52	0.75	0.51	0.25	0.46	0.36	0.70
	N	0.64	0.21	0.65	0.58	0.77	0.74	0.10	0.75	0.68	0.79	0.73	0.15	0.71	0.66	0.85	0.76	0.23	0.80	0.71	0.89
	F	0.63	0.12	0.64	0.57	0.70	0.54	0.16	0.52	0.43	0.67	0.43	0.15	0.44	0.31	0.54					
Leaf persistence																					
Persistent green	S	0.62	0.11	0.62	0.55	0.68	0.66	0.09	0.66	0.60	0.75	0.66	0.14	0.68	0.56	0.77	0.68	0.21	0.68	0.55	0.76
	N	0.65	0.15	0.68	0.58	0.75	0.68	0.11	0.67	0.63	0.71	0.77	0.09	0.77	0.74	0.82	0.78	0.23	0.79	0.66	1.00
	F	0.47	0.13	0.48	0.39	0.52	0.37	0.10	0.36	0.29	0.44	0.65	0.09	0.64	0.60	0.70					
Summer green	S	0.62	0.28	0.69	0.44	0.79	0.84	0.11	0.85	0.78	0.90	0.72	0.27	0.76	0.62	0.92	0.62	0.32	0.67	0.39	0.91
	N	0.69	0.14	0.72	0.57	0.80	0.79	0.12	0.79	0.74	0.87	0.79	0.16	0.79	0.76	0.86	0.69	0.28	0.75	0.54	0.94
	F	0.77	0.15	0.73	0.62	0.92	0.66	0.20	0.65	0.56	0.79	0.63	0.18	0.66	0.55	0.73					
Spring green	S	0.23	0.42	0.00	0.00	0.00	0.03	0.17	0.00	0.00	0.00	0.00	0.00	0.00	0.00	0.00	0.00	0.00	0.00	0.00	0.00
	N	0.24	0.35	0.05	0.00	0.34	0.26	0.44	0.00	0.00	0.54	0.00	0.00	0.00	0.00	0.00	0.00	0.00	0.00	0.00	0.00
	F	0.06	0.08	0.04	0.00	0.11	0.00	0.00	0.00	0.00	0.00	0.00	0.00	0.00	0.00	0.00					
Overwintering green	S	0.15	0.33	0.00	0.00	0.00	0.32	0.45	0.00	0.00	0.92	0.13	0.32	0.00	0.00	0.00	0.17	0.38	0.00	0.00	0.00
	N	0.10	0.29	0.00	0.00	0.00	0.06	0.24	0.00	0.00	0.00	0.00	0.00	0.00	0.00	0.00	0.00	0.00	0.00	0.00	0.00
	F	0.59	0.18	0.61	0.46	0.75	0.42	0.19	0.43	0.29	0.52	0.33	0.17	0.35	0.18	0.43					
Leaf anatomy																					
Succulent	S	0.00	0.00	0.00	0.00	0.00	0.00	0.00	0.00	0.00	0.00	0.43	0.50	0.00	0.00	1.00	0.56	0.49	1.00	0.00	1.00
	N	0.00	0.00	0.00	0.00	0.00	0.00	0.00	0.00	0.00	0.00	0.00	0.00	0.00	0.00	0.00	0.06	0.24	0.00	0.00	0.00
	F	0.00	0.00	0.00	0.00	0.00	0.00	0.00	0.00	0.00	0.00	0.00	0.00	0.00	0.00	0.00					
Succulent/hygromorphic	S	0.03	0.17	0.00	0.00	0.00	0.00	0.00	0.00	0.00	0.00	0.00	0.00	0.00	0.00	0.00	0.00	0.00	0.00	0.00	0.00

Observation time 9–12

Traits and trait states	Top.	9 Mean	SD	Med.	1st qu.	3rd qu.	10 Mean	SD	Med.	1st qu.	3rd qu.	11 Mean	SD	Med.	1st qu.	3rd qu.	12 Mean	SD	Med.	1st qu.	3rd qu.
Life span																					
Annual	S	0.28	0.42	0.00	0.00	0.54	0.00	0.01	0.00	0.00	0.00	0.00	0.00	0.00	0.00	0.00	0.00	0.00	0.00	0.00	0.00
	N	0.00	0.00	0.00	0.00	0.00	0.00	0.00	0.00	0.00	0.00	0.03	0.17	0.00	0.00	0.00	0.00	0.00	0.00	0.00	0.00
	F						0.00	0.00	0.00	0.00	0.00	0.00	0.00	0.00	0.00	0.00	0.00	0.00	0.00	0.00	0.00
Biennial	S	0.28	0.42	0.00	0.00	0.54	0.00	0.00	0.00	0.00	0.00	0.00	0.00	0.00	0.00	0.00	0.00	0.00	0.00	0.00	0.00
	N	0.00	0.00	0.00	0.00	0.00	0.03	0.17	0.00	0.00	0.00	0.00	0.00	0.00	0.00	0.00	0.00	0.00	0.00	0.00	0.00
	F						0.00	0.00	0.00	0.00	0.00	0.00	0.00	0.00	0.00	0.00	0.00	0.00	0.00	0.00	0.00
Perennial	S	0.36	0.17	0.36	0.24	0.47	0.55	0.16	0.56	0.46	0.63	0.17	0.38	0.00	0.00	0.00	0.00	0.00	0.00	0.00	0.00
	N	0.67	0.19	0.68	0.56	0.80	0.58	0.24	0.61	0.47	0.69	0.11	0.24	0.00	0.00	0.07	0.00	0.00	0.00	0.00	0.00
	F						0.39	0.47	0.01	0.00	1.00	0.05	0.17	0.00	0.00	0.00	0.00	0.00	0.00	0.00	0.00
Vegetative propagation																					
Absent	S	0.38	0.17	0.38	0.26	0.48	0.31	0.20	0.27	0.18	0.42	0.00	0.00	0.00	0.00	0.00	0.00	0.00	0.00	0.00	0.00
	N	0.64	0.19	0.64	0.52	0.75	0.51	0.27	0.46	0.26	0.70	0.14	0.28	0.00	0.00	0.13	0.00	0.00	0.00	0.00	0.00
	F						0.01	0.08	0.00	0.00	0.00	0.00	0.00	0.00	0.00	0.00	0.00	0.00	0.00	0.00	0.00
Root, tuber or bulb splitter	S	0.00	0.00	0.00	0.00	0.00	0.00	0.01	0.00	0.00	0.00	0.17	0.38	0.00	0.00	0.00	0.00	0.00	0.00	0.00	0.00
	N	0.00	0.00	0.00	0.00	0.00	0.40	0.50	0.00	0.00	1.00	0.00	0.00	0.00	0.00	0.00	0.00	0.00	0.00	0.00	0.00
	F						0.38	0.47	0.00	0.00	1.00	0.05	0.17	0.00	0.00	0.00	0.00	0.00	0.00	0.00	0.00
Bulbils	S	0.06	0.24	0.00	0.00	0.00	0.00	0.00	0.00	0.00	0.00	0.00	0.00	0.00	0.00	0.00	0.00	0.00	0.00	0.00	0.00
	N	0.10	0.29	0.00	0.00	0.00	0.03	0.17	0.00	0.00	0.00	0.00	0.00	0.00	0.00	0.00	0.00	0.00	0.00	0.00	0.00
	F						0.00	0.00	0.00	0.00	0.00	0.00	0.00	0.00	0.00	0.00	0.00	0.00	0.00	0.00	0.00
Runner / runner-like rhizome	S	0.09	0.29	0.00	0.00	0.00	0.00	0.00	0.00	0.00	0.00	0.00	0.00	0.00	0.00	0.00	0.00	0.00	0.00	0.00	0.00
	N	0.16	0.35	0.00	0.00	0.00	0.12	0.28	0.00	0.00	0.00	0.00	0.00	0.00	0.00	0.00	0.00	0.00	0.00	0.00	0.00
	F						0.00	0.00	0.00	0.00	0.00	0.00	0.00	0.00	0.00	0.00	0.00	0.00	0.00	0.00	0.00
Rhizome	S	0.05	0.11	0.00	0.00	0.00	0.00	0.00	0.00	0.00	0.00	0.00	0.00	0.00	0.00	0.00	0.00	0.00	0.00	0.00	0.00
	N	0.08	0.25	0.00	0.00	0.00	0.01	0.06	0.00	0.00	0.00	0.00	0.00	0.00	0.00	0.00	0.00	0.00	0.00	0.00	0.00
	F						0.00	0.00	0.00	0.00	0.00	0.00	0.00	0.00	0.00	0.00	0.00	0.00	0.00	0.00	0.00
Storage organs																					
Absent	S	0.00	0.00	0.00	0.00	0.00	0.00	0.01	0.00	0.00	0.00	0.00	0.00	0.00	0.00	0.00	0.00	0.00	0.00	0.00	0.00
	N	0.01	0.05	0.00	0.00	0.00	0.00	0.00	0.00	0.00	0.00	0.00	0.00	0.00	0.00	0.00	0.00	0.00	0.00	0.00	0.00
	F						0.00	0.00	0.00	0.00	0.00	0.00	0.00	0.00	0.00	0.00	0.00	0.00	0.00	0.00	0.00
Rhizome	S	0.21	0.36	0.00	0.00	0.20	0.05	0.21	0.00	0.00	0.00	0.00	0.00	0.00	0.00	0.00	0.00	0.00	0.00	0.00	0.00
	N	0.49	0.33	0.50	0.29	0.75	0.45	0.34	0.31	0.23	0.75	0.06	0.13	0.00	0.00	0.00	0.00	0.00	0.00	0.00	0.00
	F						0.00	0.00	0.00	0.00	0.00	0.00	0.00	0.00	0.00	0.00	0.00	0.00	0.00	0.00	0.00
Bulb	S	0.06	0.24	0.00	0.00	0.00	0.00	0.00	0.00	0.00	0.00	0.00	0.00	0.00	0.00	0.00	0.00	0.00	0.00	0.00	0.00
	N	0.10	0.29	0.00	0.00	0.00	0.03	0.17	0.00	0.00	0.00	0.00	0.00	0.00	0.00	0.00	0.00	0.00	0.00	0.00	0.00
	F						0.00	0.00	0.00	0.00	0.00	0.00	0.00	0.00	0.00	0.00	0.00	0.00	0.00	0.00	0.00
Tuber	S	0.00	0.01	0.00	0.00	0.00	0.00	0.00	0.00	0.00	0.00	0.17	0.38	0.00	0.00	0.00	0.00	0.00	0.00	0.00	0.00
	N	0.00	0.00	0.00	0.00	0.00	0.00	0.00	0.00	0.00	0.00	0.00	0.00	0.00	0.00	0.00	0.00	0.00	0.00	0.00	0.00
	F						0.38	0.47	0.00	0.00	1.00	0.05	0.17	0.00	0.00	0.00	0.00	0.00	0.00	0.00	0.00
Persistent tap root	S	0.37	0.16	0.37	0.27	0.47	0.52	0.18	0.55	0.45	0.63	0.00	0.00	0.00	0.00	0.00	0.00	0.00	0.00	0.00	0.00
	N	0.71	0.26	0.71	0.54	0.98	0.55	0.37	0.57	0.19	1.00	0.12	0.32	0.00	0.00	0.00	0.00	0.00	0.00	0.00	0.00
	F						0.02	0.08	0.00	0.00	0.00	0.00	0.00	0.00	0.00	0.00	0.00	0.00	0.00	0.00	0.00
Leaf persistence																					
Persistent green	S	0.31	0.17	0.33	0.20	0.42	0.19	0.19	0.13	0.03	0.32	0.00	0.00	0.00	0.00	0.00	0.00	0.00	0.00	0.00	0.00
	N	0.46	0.42	0.47	0.00	0.96	0.23	0.32	0.05	0.00	0.50	0.00	0.00	0.00	0.00	0.00	0.00	0.00	0.00	0.00	0.00
	F						0.00	0.01	0.00	0.00	0.00	0.00	0.00	0.00	0.00	0.00	0.00	0.00	0.00	0.00	0.00
Summer green	S	0.42	0.32	0.38	0.17	0.64	0.73	0.31	0.83	0.57	1.00	0.17	0.38	0.00	0.00	0.00	0.00	0.00	0.00	0.00	0.00
	N	0.60	0.30	0.60	0.43	0.80	0.61	0.30	0.63	0.42	0.89	0.14	0.28	0.00	0.00	0.13	0.00	0.00	0.00	0.00	0.00
	F						0.39	0.47	0.00	0.00	1.00	0.05	0.17	0.00	0.00	0.00	0.00	0.00	0.00	0.00	0.00
Spring green	S	0.00	0.00	0.00	0.00	0.00	0.00	0.00	0.00	0.00	0.00	0.00	0.00	0.00	0.00	0.00	0.00	0.00	0.00	0.00	0.00
	N	0.00	0.00	0.00	0.00	0.00	0.00	0.00	0.00	0.00	0.00	0.00	0.00	0.00	0.00	0.00	0.00	0.00	0.00	0.00	0.00
	F						0.00	0.00	0.00	0.00	0.00	0.00	0.00	0.00	0.00	0.00	0.00	0.00	0.00	0.00	0.00
Overwintering green	S	0.07	0.25	0.00	0.00	0.00	0.00	0.00	0.00	0.00	0.00	0.00	0.00	0.00	0.00	0.00	0.00	0.00	0.00	0.00	0.00
	N	0.06	0.24	0.00	0.00	0.00	0.03	0.17	0.00	0.00	0.00	0.00	0.00	0.00	0.00	0.00	0.00	0.00	0.00	0.00	0.00
	F						0.00	0.00	0.00	0.00	0.00	0.00	0.00	0.00	0.00	0.00	0.00	0.00	0.00	0.00	0.00
Leaf anatomy																					
Succulent	S	0.07	0.24	0.00	0.00	0.00	0.00	0.00	0.00	0.00	0.00	0.00	0.00	0.00	0.00	0.00	0.00	0.00	0.00	0.00	0.00
	N	0.00	0.00	0.00	0.00	0.00	0.00	0.00	0.00	0.00	0.00	0.00	0.00	0.00	0.00	0.00	0.00	0.00	0.00	0.00	0.00
	F						0.00	0.00	0.00	0.00	0.00	0.00	0.00	0.00	0.00	0.00	0.00	0.00	0.00	0.00	0.00
Succulent/hygromorphic	S	0.00	0.00	0.00	0.00	0.00	0.00	0.00	0.00	0.00	0.00	0.00	0.00	0.00	0.00	0.00	0.00	0.00	0.00	0.00	0.00

	N	0.00	0.00	0.00	0.00	0.00	0.03	0.17	0.00	0.00	0.00	0.16	0.26	0.10	0.00	0.15	0.68	0.34	0.82	0.56	0.95	0.16	0.27	0.00	0.00	0.21	0.00	0.01	0.00	0.00	0.00	0.00	0.00	0.00	0.00	0.00	0.00	0.00	0.00	0.00	0.00	0.00	0.00	0.00	0.00	0.00	0.00	0.00	0.00	0.00	0.00	0.00	0.00	0.00	0.00	0.00	0.00	0.00	0.00	0.00	0.00
	F	0.00	0.00	0.00	0.00	0.00	0.00	0.00	0.00	0.00	0.00	0.00	0.00	0.00	0.00	0.00	0.03	0.17	0.00	0.00	0.00	0.00	0.00	0.00	0.00	0.00	0.00	0.00	0.00	0.00	0.00	0.00	0.00	0.00	0.00	0.00											0.00	0.00	0.00	0.00	0.00	0.00	0.00	0.00	0.00	0.00	0.00	0.00	0.00	0.00	0.00
Hygromorphic	S	0.00	0.00	0.00	0.00	0.00	0.00	0.00	0.00	0.00	0.00	0.00	0.00	0.00	0.00	0.00	0.00	0.00	0.00	0.00	0.00	0.00	0.00	0.00	0.00	0.00	0.00	0.00	0.00	0.00	0.00	0.00	0.00	0.00	0.00	0.00	0.00	0.00	0.00	0.00	0.00	0.00	0.00	0.00	0.00	0.00	0.00	0.00	0.00	0.00	0.00	0.00	0.00	0.00	0.00	0.00	0.00	0.00	0.00	0.00	0.00
	N	0.03	0.17	0.00	0.00	0.00	0.00	0.00	0.00	0.00	0.00	0.00	0.00	0.00	0.00	0.00	0.00	0.00	0.00	0.00	0.00	0.00	0.00	0.00	0.00	0.00	0.00	0.00	0.00	0.00	0.00	0.09	0.28	0.00	0.00	0.00	0.00	0.00	0.00	0.00	0.00	0.00	0.00	0.00	0.00	0.00	0.00	0.00	0.00	0.00	0.00	0.00	0.00	0.00	0.00	0.00	0.00	0.00	0.00	0.00	0.00
	F	0.00	0.00	0.00	0.00	0.00	0.06	0.24	0.00	0.00	0.00	0.00	0.00	0.00	0.00	0.00	0.02	0.10	0.00	0.00	0.00	0.80	0.38	1.00	0.94	1.00	0.04	0.17	0.00	0.00	0.00	0.00	0.00	0.00	0.00	0.00											0.00	0.00	0.00	0.00	0.00	0.00	0.00	0.00	0.00	0.00	0.00	0.00	0.00	0.00	0.00
Mesomorphic	S	0.97	0.17	1.00	1.00	1.00	0.60	0.28	0.56	0.43	0.77	0.73	0.19	0.75	0.61	0.86	0.60	0.32	0.69	0.45	0.79	0.67	0.20	0.62	0.52	0.88	0.82	0.13	0.84	0.77	0.92	0.79	0.23	0.85	0.73	1.00	0.35	0.46	0.00	0.00	1.00	0.28	0.41	0.01	0.00	0.42	0.74	0.44	1.00	0.52	1.00	0.17	0.38	0.00	0.00	0.00	0.00	0.00	0.00	0.00	0.00
	N	0.49	0.35	0.35	0.19	0.82	0.56	0.28	0.52	0.38	0.76	0.58	0.18	0.59	0.46	0.71	0.72	0.15	0.72	0.66	0.83	0.72	0.15	0.73	0.64	0.82	0.67	0.17	0.68	0.60	0.77	0.77	0.14	0.78	0.68	0.87	0.73	0.34	0.82	0.63	1.00	0.33	0.40	0.14	0.00	0.55	0.20	0.33	0.00	0.00	0.27	0.03	0.17	0.00	0.00	0.00	0.00	0.00	0.00	0.00	0.00
	F	0.98	0.05	1.00	1.00	1.00	0.08	0.15	0.02	0.01	0.07	0.60	0.14	0.58	0.54	0.70	0.54	0.13	0.52	0.43	0.63	0.50	0.08	0.50	0.44	0.57	0.42	0.09	0.41	0.35	0.47	0.56	0.07	0.56	0.53	0.59											0.39	0.47	0.01	0.00	1.00	0.05	0.17	0.00	0.00	0.00	0.00	0.00	0.00	0.00	0.00
Mesomorphic/hygromorphic	S	0.00	0.00	0.00	0.00	0.00	0.01	0.03	0.00	0.00	0.00	0.70	0.25	0.71	0.62	0.86	0.51	0.31	0.42	0.29	0.72	0.00	0.00	0.00	0.00	0.00	0.00	0.00	0.00	0.00	0.00	0.00	0.00	0.00	0.00	0.00	0.00	0.00	0.00	0.00	0.00	0.06	0.24	0.00	0.00	0.00	0.00	0.00	0.00	0.00	0.00	0.00	0.00	0.00	0.00	0.00	0.00	0.00	0.00	0.00	0.00
	N	0.00	0.00	0.00	0.00	0.00	0.15	0.33	0.00	0.00	0.00	0.22	0.36	0.00	0.00	0.32	0.75	0.30	0.83	0.64	1.00	0.53	0.39	0.67	0.17	1.00	0.24	0.40	0.00	0.00	0.42	0.00	0.00	0.00	0.00	0.00	0.00	0.00	0.00	0.00	0.00	0.06	0.24	0.00	0.00	0.00	0.03	0.17	0.00	0.00	0.00	0.00	0.00	0.00	0.00	0.00	0.00	0.00	0.00	0.00	0.00
	F	0.04	0.07	0.00	0.00	0.05	0.27	0.26	0.15	0.07	0.42	0.23	0.25	0.11	0.04	0.34	0.40	0.32	0.40	0.07	0.69	0.52	0.30	0.52	0.36	0.67	0.74	0.30	0.86	0.52	0.98	0.02	0.06	0.00	0.00	0.02											0.00	0.00	0.00	0.00	0.00	0.00	0.00	0.00	0.00	0.00	0.00	0.00	0.00	0.00	0.00
Scleromorphic	S	0.00	0.00	0.00	0.00	0.00	0.32	0.42	0.00	0.00	0.70	0.14	0.36	0.00	0.00	0.00	0.28	0.34	0.06	0.00	0.52	0.49	0.21	0.51	0.34	0.59	0.69	0.20	0.75	0.59	0.87	0.62	0.20	0.60	0.54	0.72	0.60	0.20	0.57	0.50	0.74	0.43	0.22	0.45	0.27	0.58	0.28	0.18	0.27	0.15	0.42	0.00	0.00	0.00	0.00	0.00	0.00	0.00	0.00	0.00	0.00
	N	0.14	0.36	0.00	0.00	0.00	0.19	0.39	0.00	0.00	0.00	0.07	0.25	0.00	0.00	0.00	0.31	0.44	0.00	0.00	0.85	0.37	0.45	0.00	0.00	1.00	0.70	0.26	0.74	0.59	0.86	0.71	0.24	0.75	0.60	0.88	0.74	0.40	1.00	0.61	1.00	0.29	0.44	0.00	0.00	0.79	0.51	0.50	0.88	0.00	1.00	0.09	0.28	0.00	0.00	0.00	0.00	0.00	0.00	0.00	0.00
	F	0.21	0.40	0.00	0.00	0.00	0.12	0.31	0.00	0.00	0.00	0.07	0.25	0.00	0.00	0.00	0.00	0.00	0.00	0.00	0.00	0.00	0.00	0.00	0.00	0.00	0.09	0.28	0.00	0.00	0.00	0.26	0.44	0.00	0.00	0.50											0.00	0.00	0.00	0.00	0.00	0.00	0.00	0.00	0.00	0.00	0.00	0.00	0.00	0.00	0.00
Scleromorphic/mesomorphic	S	0.00	0.00	0.00	0.00	0.00	0.54	0.40	0.61	0.05	0.97	0.77	0.19	0.77	0.66	1.00	0.64	0.17	0.65	0.52	0.77	0.69	0.15	0.69	0.66	0.77	0.66	0.09	0.65	0.62	0.72	0.67	0.21	0.71	0.52	0.82	0.10	0.29	0.00	0.00	0.00	0.12	0.21	0.03	0.00	0.11	0.04	0.18	0.00	0.00	0.00	0.00	0.00	0.00	0.00	0.00	0.00	0.00	0.00	0.00	0.00
	N	0.00	0.00	0.00	0.00	0.00	0.16	0.29	0.00	0.00	0.17	0.55	0.30	0.61	0.40	0.74	0.68	0.18	0.68	0.55	0.85	0.60	0.19	0.60	0.49	0.71	0.73	0.12	0.72	0.66	0.81	0.79	0.14	0.80	0.72	0.89	0.56	0.30	0.53	0.30	0.72	0.63	0.22	0.62	0.51	0.75	0.47	0.32	0.33	0.23	0.75	0.06	0.13	0.00	0.00	0.00	0.00	0.00	0.00	0.00	0.00
	F	0.00	0.00	0.00	0.00	0.00	0.51	0.48	0.67	0.00	1.00	0.59	0.46	0.83	0.00	1.00	0.69	0.37	0.96	0.38	1.00	0.60	0.25	0.57	0.42	0.84	0.59	0.21	0.64	0.47	0.73	0.58	0.28	0.68	0.49	0.76											0.00	0.00	0.00	0.00	0.00	0.00	0.00	0.00	0.00	0.00	0.00	0.00	0.00	0.00	0.00
Horizontal space occupation																																																													
Absent	S	0.00	0.00	0.00	0.00	0.00	0.53	0.35	0.58	0.27	0.78	0.84	0.13	0.87	0.74	0.94	0.58	0.25	0.57	0.40	0.75	0.61	0.32	0.61	0.50	0.88	0.78	0.18	0.76	0.64	0.95	0.72	0.24	0.71	0.64	0.88	0.68	0.28	0.75	0.49	0.91	0.36	0.27	0.31	0.17	0.50	0.18	0.24	0.03	0.00	0.33	0.00	0.00	0.00	0.00	0.00	0.00	0.00	0.00	0.00	0.00
	N	0.14	0.36	0.00	0.00	0.00	0.28	0.43	0.00	0.00	0.71	0.59	0.32	0.61	0.41	0.78	0.83	0.13	0.84	0.77	0.92	0.68	0.23	0.73	0.54	0.83	0.79	0.22	0.80	0.66	1.00	0.68	0.28	0.68	0.53	0.96	0.81	0.27	0.88	0.68	1.00	0.48	0.45	0.48	0.00	1.00	0.37	0.43	0.10	0.00	0.94	0.12	0.32	0.00	0.00	0.00	0.00	0.00	0.00	0.00	0.00
	F	0.21	0.40	0.00	0.00	0.00	0.12	0.31	0.00	0.00	0.00	0.14	0.31	0.01	0.00	0.06	0.59	0.15	0.53	0.49	0.67	0.60	0.13	0.57	0.53	0.71	0.52	0.16	0.50	0.43	0.60	0.36	0.19	0.37	0.24	0.48											0.01	0.08	0.00	0.00	0.00	0.00	0.00	0.00	0.00	0.00	0.00	0.00	0.00	0.00	0.00
Caespitose	S	0.00	0.00	0.00	0.00	0.00	0.17	0.38	0.00	0.00	0.00	0.14	0.34	0.00	0.00	0.00	0.64	0.34	0.73	0.47	0.96	0.62	0.39	0.81	0.30	0.99	0.83	0.13	0.84	0.73	0.96	0.32	0.38	0.03	0.00	0.70	0.00	0.00	0.00	0.00	0.00	0.08	0.18	0.00	0.00	0.09	0.00	0.00	0.00	0.00	0.00	0.00	0.00	0.00	0.00	0.00	0.00	0.00	0.00	0.00	0.00
	N	0.00	0.00	0.00	0.00	0.00	0.00	0.01	0.00	0.00	0.00	0.10	0.17	0.00	0.00	0.13	0.45	0.32	0.49	0.18	0.69	0.72	0.25	0.75	0.64	0.94	0.49	0.24	0.46	0.35	0.71	0.77	0.16	0.78	0.75	0.84	0.02	0.09	0.00	0.00	0.00	0.02	0.06	0.00	0.00	0.00	0.00	0.01	0.00	0.00	0.00	0.00	0.00	0.00	0.00	0.00	0.00	0.00	0.00	0.00	0.00
	F	0.00	0.00	0.00	0.00	0.00	0.01	0.01	0.01	0.00	0.02	0.34	0.19	0.28	0.21	0.47	0.57	0.25	0.61	0.40	0.76	0.30	0.13	0.29	0.23	0.37	0.36	0.12	0.36	0.29	0.42	0.82	0.09	0.82	0.78	0.87											0.00	0.00	0.00	0.00	0.00	0.00	0.00	0.00	0.00	0.00	0.00	0.00	0.00	0.00	0.00
Pleiocorm	S	0.00	0.00	0.00	0.00	0.00	0.20	0.26	0.14	0.02	0.23	0.52	0.34	0.60	0.22	0.81	0.58	0.23	0.58	0.41	0.75	0.60	0.16	0.60	0.47	0.71	0.69	0.12	0.69	0.62	0.80	0.68	0.14	0.65	0.57	0.80	0.45	0.32	0.51	0.15	0.64	0.47	0.32	0.46	0.18	0.72	0.58	0.27	0.57	0.53	0.72	0.00	0.00	0.00	0.00	0.00	0.00	0.00	0.00	0.00	0.00
	N	0.00	0.00	0.00	0.00	0.00	0.04	0.17	0.00	0.00	0.00	0.40	0.25	0.36	0.21	0.60	0.71	0.19	0.75	0.63	0.82	0.61	0.21	0.64	0.51	0.76	0.74	0.16	0.75	0.67	0.83	0.80	0.15	0.79	0.71	0.96	0.69	0.25	0.67	0.56	0.91	0.67	0.25	0.73	0.53	0.83	0.58	0.31	0.58	0.32	0.89	0.06	0.13	0.00	0.00	0.00	0.00	0.00	0.00	0.00	0.00
	F	0.00	0.00	0.00	0.00	0.00	0.09	0.22	0.00	0.00	0.04	0.91	0.18	1.00	0.92	1.00	0.23	0.23	0.14	0.08	0.29	0.61	0.16	0.64	0.52	0.75	0.54	0.16	0.53	0.44	0.68	0.40	0.14	0.44	0.30	0.50											0.00	0.01	0.00	0.00	0.00	0.00	0.00	0.00	0.00	0.00	0.00	0.00	0.00	0.00	0.00
Reptant	S	0.00	0.00	0.00	0.00	0.00	0.53	0.45	0.67	0.00	1.00	0.51	0.43	0.54	0.02	1.00	0.58	0.33	0.64	0.33	0.84	0.60	0.20	0.63	0.46	0.74	0.64	0.19	0.67	0.54	0.73	0.64	0.38	0.68	0.43	1.00	0.51	0.50	0.70	0.00	1.00	0.07	0.20	0.00	0.00	0.01	0.00	0.00	0.00	0.00	0.00	0.00	0.00	0.00	0.00	0.00	0.00	0.00	0.00	0.00	0.00
	N	0.07	0.19	0.00	0.00	0.00	0.35	0.39	0.20	0.00	0.68	0.63	0.27	0.69	0.57	0.77	0.62	0.23	0.67	0.49	0.77	0.61	0.25	0.60	0.47	0.76	0.73	0.19	0.72	0.62	0.87	0.79	0.19	0.81	0.72	0.88	0.42	0.43	0.25	0.00	1.00	0.15	0.36	0.00	0.00	0.00	0.12	0.28	0.00	0.00	0.00	0.00	0.00	0.00	0.00	0.00	0.00	0.00	0.00	0.00	0.00
	F	0.00	0.00	0.00	0.00	0.00	0.37	0.49	0.00	0.00	1.00	0.46	0.51	0.00	0.00	1.00	0.56	0.50	1.00	0.00	1.00	0.41	0.45	0.17	0.00	0.97	0.28	0.39	0.00	0.00	0.61	0.80	0.34	1.00	0.80	1.00											0.00	0.00	0.00	0.00	0.00	0.00	0.00	0.00	0.00	0.00	0.00	0.00	0.00	0.00	0.00
Rosulate	S	0.97	0.17	1.00	1.00	1.00	0.55	0.39	0.52	0.24	1.00	0.69	0.30	0.73	0.55	1.00	0.64	0.31	0.70	0.57	0.83	0.47	0.41	0.40	0.00	0.95	0.58	0.41	0.67	0.00	1.00	0.68	0.40	0.86	0.53	1.00	0.06	0.24	0.00	0.00	0.00	0.00	0.00	0.00	0.00	0.00	0.00	0.00	0.00	0.00	0.00	0.17	0.38	0.00	0.00	0.00	0.00	0.00	0.00	0.00	0.00
	N	0.44	0.38	0.29	0.12	0.82	0.47	0.33	0.50	0.20	0.69	0.55	0.28	0.55	0.36	0.77	0.52	0.42	0.67	0.00	0.95	0.55	0.45	0.67	0.00	1.00	0.74	0.34	0.90	0.59	1.00	0.46	0.48	0.33	0.00	1.00	0.08	0.26	0.00	0.00	0.00	0.06	0.19	0.00	0.00	0.00	0.02	0.07	0.00	0.00	0.00	0.00	0.00	0.00	0.00	0.00	0.00	0.00	0.00	0.00	0.00
	F	0.77	0.23	0.86	0.53	1.00	0.36	0.26	0.39	0.14	0.56	0.57	0.25	0.61	0.45	0.73	0.58	0.30	0.68	0.42	0.81	0.68	0.36	0.78	0.52	1.00	0.15	0.30	0.00	0.00	0.10	0.03	0.08	0.00	0.00	0.02											0.38	0.47	0.00	0.00	1.00	0.05	0.17	0.00	0.00	0.00	0.00	0.00	0.00	0.00	0.00
Climber	S	0.00	0.00	0.00	0.00	0.00	0.00	0.00	0.00	0.00	0.00	0.00	0.00	0.00	0.00	0.00	0.00	0.00	0.00	0.00	0.00	0.00	0.00	0.00	0.00	0.00	0.00	0.00	0.00	0.00	0.00	0.00	0.00	0.00	0.00	0.00	0.00	0.00	0.00	0.00	0.00	0.00	0.00	0.00	0.00	0.00	0.00	0.00	0.00	0.00	0.00	0.00	0.00	0.00	0.00	0.00	0.00	0.00	0.00	0.00	0.00
	N	0.00	0.00	0.00	0.00	0.00	0.00	0.00	0.00	0.00	0.00	0.00	0.00	0.00	0.00	0.00	0.00	0.00	0.00	0.00	0.00	0.00	0.00	0.00	0.00	0.00	0.00	0.00	0.00	0.00	0.00	0.00	0.00	0.00	0.00	0.00	0.03	0.17	0.00	0.00	0.00	0.00	0.00	0.00	0.00	0.00	0.00	0.00	0.00	0.00	0.00	0.00	0.00	0.00	0.00	0.00	0.00	0.00	0.00	0.00	0.00
	F	0.00	0.00	0.00	0.00	0.00	0.00	0.00	0.00	0.00	0.00	0.00	0.00	0.00	0.00	0.00	0.00	0.00	0.00	0.00	0.00	0.09	0.28	0.00	0.00	0.00	0.03	0.17	0.00	0.00	0.00	0.00	0.00	0.00	0.00	0.00											0.00	0.00	0.00	0.00	0.00	0.00	0.00	0.00	0.00	0.00	0.00	0.00	0.00	0.00	0.00
Prostrate	S	0.00	0.00	0.00	0.00	0.00	0.00	0.00	0.00	0.00	0.00	0.01	0.07	0.00	0.00	0.00	0.12	0.31	0.00	0.00	0.00	0.17	0.37	0.00	0.00	0.00	0.10	0.28	0.00	0.00	0.00	0.00	0.00	0.00	0.00	0.00	0.00	0.00	0.00	0.00	0.00	0.00	0.00	0.00	0.00	0.00	0.00	0.00	0.00	0.00	0.00	0.00	0.00	0.00	0.00	0.00	0.00	0.00	0.00	0.00	0.00
	N	0.00	0.00	0.00	0.00	0.00	0.00	0.00	0.00	0.00	0.00	0.00	0.03	0.00	0.00	0.00	0.05	0.22	0.00	0.00	0.00	0.00	0.00	0.00	0.00	0.00	0.00	0.00	0.00	0.00	0.00	0.00	0.00	0.00	0.00	0.00	0.00	0.00	0.00	0.00	0.00	0.00	0.00	0.00	0.00	0.00	0.00	0.00	0.00	0.00	0.00	0.00	0.00	0.00	0.00	0.00	0.00	0.00	0.00	0.00	0.00
	F	0.00	0.00	0.00	0.00	0.00	0.06	0.24	0.00	0.00	0.00	0.00	0.00	0.00	0.00	0.00	0.11	0.29	0.00	0.00	0.00	0.37	0.45	0.00	0.00	0.96	0.11	0.26	0.00	0.00	0.04	0.50	0.44	0.60	0.00	0.95											0.00	0.00	0.00	0.00	0.00	0.00	0.00	0.00	0.00	0.00	0.00	0.00	0.00	0.00	0.00
Vertical space occupation																																																													
No leafy stem with narrow basal leaves	S	0.00	0.00	0.00	0.00	0.00	0.18	0.33	0.00	0.00	0.23	0.43	0.42	0.33	0.00	0.91	0.50	0.39	0.60	0.02	0.80	0.00	0.02	0.00	0.00	0.00	0.00	0.00	0.00	0.00	0.00	0.00	0.00	0.00	0.00	0.00	0.00	0.00	0.00	0.00	0.00	0.00	0.00	0.00	0.00	0.00	0.00	0.00	0.00	0.00	0.00	0.00	0.00	0.00	0.00	0.00	0.00	0.00	0.00	0.00	0.00
	N	0.09	0.28	0.00	0.00	0.00	0.00	0.00	0.00	0.00	0.00	0.03	0.17	0.00	0.00	0.00	0.09	0.28	0.00	0.00	0.00	0.00	0.00	0.00	0.00	0.00	0.14	0.36	0.00	0.00	0.00	0.00	0.00	0.00	0.00	0.00	0.00	0.00	0.00	0.00	0.00	0.00	0.00	0.00	0.00	0.00	0.00	0.00	0.00	0.00	0.00	0.00	0.00	0.00	0.00	0.00	0.00	0.00	0.00	0.00	0.00
	F	0.98	0.05	1.00	1.00	1.00	0.02	0.05	0.00	0.00	0.00	0.00	0.00	0.00	0.00	0.00	0.09	0.28	0.00	0.00	0.00	0.66	0.48	1.00	0.00	1.00	0.00	0.00	0.00	0.00	0.00	0.00	0.00	0.00	0.00	0.00											0.00	0.00	0.00	0.00	0.00	0.00	0.00	0.00	0.00	0.00	0.00	0.00	0.00	0.00	0.00
Grass	S	0.00	0.00	0.00	0.00	0.00	0.03	0.17	0.00	0.00	0.00	0.05	0.21	0.00	0.00	0.00	0.26	0.41	0.00	0.00	0.40	0.36	0.48	0.00	0.00	1.00	0.91	0.14	1.00	0.81	1.00	0.68	0.25	0.73	0.61	0.83	0.16	0.17	0.13	0.00	0.24	0.33	0.27	0.29	0.09	0.49	0.19	0.19	0.13	0.02	0.32	0.00	0.00	0.00	0.00	0.00	0.00	0.00	0.00	0.00	0.00
	N	0.00	0.00	0.00	0.00	0.00	0.00	0.01	0.00	0.00	0.00	0.09	0.15	0.00	0.00	0.13	0.34	0.32	0.25	0.00	0.65	0.72	0.26	0.73	0.57	0.94	0.52	0.31	0.50	0.30	0.82	0.79	0.17	0.81	0.75	0.85	0.27	0.37	0.00	0.00	0.56	0.59	0.44	0.80	0.00	1.00	0.14	0.29	0.00	0.00	0.17	0.00	0.00	0.00	0.00	0.00	0.00	0.00	0.00	0.00	0.00
	F	0.00	0.00	0.00	0.00	0.00	0.01	0.01	0.01	0.00	0.02	0.34	0.19	0.28	0.21	0.47	0.57	0.25	0.61	0.40	0.76	0.34	0.12	0.34	0.27	0.41	0.37	0.12	0.38	0.32	0.44	0.77	0.09	0.75	0.71	0.83											0.00	0.00	0.00	0.00	0.00	0.00	0.00	0.00	0.00	0.00	0.00	0.00	0.00	0.00	0.00
No leafy stem, with broad basal leaves (rosette forb)	S	0.97	0.17	1.00	1.00	1.00	0.44	0.40	0.50	0.00	0.75	0.68	0.35	0.75	0.44	1.00	0.58	0.39	0.71	0.15	0.91	0.44	0.41	0.40	0.00	0.77	0.58	0.41	0.67	0.00	1.00	0.54	0.43	0.67	0.00	1.00	0.03	0.17	0.00	0.00	0.00	0.00	0.00	0.00	0.00	0.00	0.00	0.00	0.00	0.00	0.00	0.17	0.38	0.00	0.00	0.00	0.00	0.00	0.00	0.00	0.00
	N	0.39	0.38	0.24	0.10	0.72	0.47	0.35	0.50	0.16	0.76	0.46	0.31	0.32	0.22	0.71	0.50	0.44	0.60	0.00	1.00	0.55	0.45	0.67	0.00	1.00	0.71	0.36	0.83	0.44	1.00	0.46	0.48	0.33	0.00	1.00	0.08	0.26	0.00	0.00	0.00	0.06	0.19	0.00	0.00	0.00	0.02	0.07	0.00	0.00	0.00	0.00	0.00	0.00	0.00	0.00	0.00	0.00	0.00	0.00	0.00
	F	0.04	0.07	0.00	0.00	0.05	0.37	0.27	0.41	0.11	0.57	0.57	0.25	0.61	0.45	0.73	0.57	0.30	0.67	0.42	0.81	0.26	0.35	0.05	0.00	0.48	0.15	0.30	0.00	0.00	0.10	0.03	0.08	0.00	0.00	0.02											0.38	0.47	0.00	0.00	1.00	0.05	0.17	0.00	0.00	0.00	0.00	0.00	0.00	0.00	0.00
Hemirosulate upright forb	S	0.00	0.00	0.00	0.00	0.00	0.53	0.28	0.57	0.32	0.67	0.79	0.10	0.80	0.73	0.85	0.64	0.21	0.64	0.49	0.81	0.61	0.26	0.58	0.48	0.77	0.81	0.14	0.83	0.71	0.93	0.71	0.32	0.82	0.51	1.00	0.58	0.32	0.55	0.33	0.83	0.32	0.27	0.33	0.08	0.50	0.23	0.29	0.13	0.00	0.38	0.00	0.00	0.00	0.00	0.00	0.00	0.00	0.00	0.00	0.00
	N	0.07	0.19	0.00	0.00	0.00	0.39	0.36	0.38	0.04	0.62	0.60	0.15	0.62	0.51	0.68	0.74	0.11	0.75	0.67	0.82	0.62	0.15	0.60	0.55	0.69	0.75	0.19	0.83	0.60	0.90	0.82	0.13	0.82	0.75	0.89	0.67	0.21	0.70	0.57	0.80	0.66	0.23	0.65	0.47	0.85	0.63	0.25	0.63	0.55	0.75	0.14	0.28	0.00	0.00	0.13	0.00	0.00	0.00	0.00	0.00
	F	0.00	0.00	0.00	0.00	0.00	0.32	0.43	0.01	0.00	0.68	0.79	0.21	0.88	0.55	1.00	0.52	0.13	0.52	0.43	0.57	0.63	0.11	0.63	0.57	0.68	0.55	0.11	0.55	0.49	0.63	0.38	0.15	0.38	0.27	0.48											0.02	0.08	0.00	0.00	0.00	0.00	0.00	0.00	0.00	0.00	0.00	0.00	0.00	0.00	0.00
Erosulate upright forb	S	0.00	0.00	0.00	0.00	0.00	0.32	0.42	0.00	0.00	0.70	0.14	0.33	0.00	0.00	0.02	0.52	0.22	0.51	0.41	0.67	0.65	0.13	0.65	0.55	0.74	0.59	0.11	0.58	0.53	0.68	0.71	0.19	0.73	0.60	0.82	0.87	0.21	0.96	0.82	1.00	0.23	0.24	0.13	0.04	0.41	0.73	0.44	1.00	0.26	1.00	0.00	0.00	0.00	0.00	0.00	0.00	0.00	0.00	0.00	0.00
	N	0.14	0.36	0.00	0.00	0.00	0.19	0.39	0.00	0.00	0.00	0.16	0.36	0.00	0.00	0.00	0.55	0.38	0.65	0.20	0.89	0.67	0.25	0.72	0.53	0.83	0.72	0.16	0.77	0.60	0.83	0.68	0.25	0.70	0.57	0.89	0.75	0.39	1.00	0.61	1.00	0.12	0.29	0.00	0.00	0.03	0.03	0.17	0.00	0.00	0.00	0.00	0.00	0.00	0.00	0.00	0.00	0.00	0.00	0.00	0.00
	F	0.21	0.40	0.00	0.00	0.00	0.12	0.31	0.00	0.00	0.00	0.01	0.08	0.00	0.00	0.00	0.55	0.30	0.58	0.37	0.80	0.48	0.31	0.56	0.22	0.71	0.50	0.25	0.55	0.36	0.66	0.48	0.30	0.46	0.28	0.69											0.00	0.00	0.00	0.00	0.00	0.00	0.00	0.00	0.00	0.00	0.00	0.00	0.00	0.00	0.00
Climber	S	0.00	0.00	0.00	0.00	0.00	0.00	0.00	0.00	0.00	0.00	0.00	0.00	0.00	0.00	0.00	0.00	0.00	0.00	0.00	0.00	0.00	0.00	0.00	0.00	0.00	0.00	0.00	0.00	0.00	0.00	0.00	0.00	0.00	0.00	0.00	0.00	0.00	0.00	0.00	0.00	0.00	0.00	0.00	0.00	0.00	0.00	0.00	0.00	0.00	0.00	0.00	0.00	0.00	0.00	0.00	0.00	0.00	0.00	0.00	0.00
	N	0.00	0.00	0.00	0.00	0.00	0.00	0.00	0.00	0.00	0.00	0.00	0.00	0.00	0.00	0.00	0.00	0.00	0.00	0.00	0.00	0.00	0.00	0.00	0.00	0.00	0.00	0.00	0.00	0.00	0.00	0.00	0.00	0.00	0.00	0.00	0.03	0.17	0.00	0.00	0.00	0.00	0.00	0.00	0.00	0.00	0.00	0.00	0.00	0.00	0.00	0.00	0.00	0.00	0.00	0.00	0.00	0.00	0.00	0.00	0.00
	F	0.00	0.00	0.00	0.00	0.00	0.00	0.00	0.00	0.00	0.00	0.00	0.00	0.00	0.00	0.00	0.00	0.00	0.00	0.00	0.00	0.09	0.28	0.00	0.00	0.00	0.03	0.17	0.00	0.00	0.00	0.00	0.00	0.00	0.00	0.00											0.00	0.00	0.00	0.00	0.00	0.00	0.00	0.00	0.00	0.00	0.00	0.00	0.00	0.00	0.00
Prostrate forb	S	0.00	0.00	0.00	0.00	0.00	0.00	0.00	0.00	0.00	0.00	0.01	0.04	0.00	0.00	0.00	0.10	0.28	0.00	0.00	0.00	0.34	0.28	0.30	0.09	0.52	0.71	0.23	0.71	0.59	0.91	0.05	0.10	0.00	0.00	0.05	0.00	0.00	0.00	0.00	0.00	0.29	0.43	0.00	0.00	0.66	0.00	0.00	0.00	0.00	0.00	0.00	0.00	0.00	0.00	0.00	0.00	0.00	0.00	0.00	0.00
	N	0.00	0.00	0.00	0.00	0.00	0.00	0.00	0.00	0.00	0.00	0.00	0.00	0.00	0.00	0.00	0.04	0.19	0.00	0.00	0.00	0.25	0.41	0.00	0.00	0.49	0.56	0.45	0.75	0.00	1.00	0.35	0.42	0.08	0.00	0.66	0.00	0.00	0.00	0.00	0.00	0.00	0.00	0.00	0.00	0.00	0.00	0.00	0.00	0.00	0.00	0.00	0.00	0.00	0.00	0.00	0.00	0.00	0.00	0.00	0.00
	F	0.00	0.00	0.00	0.00	0.00	0.06	0.24	0.00	0.00	0.00	0.00	0.00	0.00	0.00	0.00	0.11	0.29	0.00	0.00	0.00	0.24	0.39	0.00	0.00	0.51	0.11	0.26	0.00	0.00	0.04	0.50	0.44	0.60	0.00	0.95											0.00	0.00	0.00	0.00	0.00	0.00	0.00	0.00	0.00	0.00	0.00	0.00	0.00	0.00	0.00
Plant height (cm)																																																													
≤ 20	S	0.97	0.17	1.00	1.00	1.00	0.62	0.19	0.59	0.50	0.76	0.74	0.13	0.75	0.70	0.80	0.55	0.14	0.57	0.43	0.67	0.56	0.14	0.56	0.48	0.62	0.65	0.10	0.64	0.56	0.70	0.62	0.14	0.61	0.53	0.71	0.64	0.24	0.60	0.44	0.77	0.39	0.25	0.38	0.21	0.57	0.53	0.28	0.57	0.51	0.69	0.17	0.38	0.00	0.00	0.00	0.00	0.00	0.00	0.00	0.00
	N	0.44	0.38	0.29	0.12	0.82	0.53	0.29	0.53	0.27	0.71	0.51	0.13	0.49	0.43	0.59	0.70	0.12	0.71	0.61	0.81	0.59	0.18	0.58	0.46	0.72	0.78	0.18	0.78	0.70	0.93	0.46	0.33	0.58	0.13	0.67	0.77	0.39	1.00	0.72	1.00	0.14	0.33	0.00	0.00	0.00	0.39	0.49	0.00	0.00	1.00	0.00	0.00	0.00	0.00	0.00	0.00	0.00	0.00	0.00	0.00
	F	0.77	0.23	0.86	0.53	1.00	0.44	0.23	0.49	0.32	0.58	0.73	0.16	0.71	0.62	0.83	0.55	0.17	0.52	0.42	0.69	0.59	0.18	0.62	0.51	0.69	0.42	0.14	0.43	0.33	0.52	0.33	0.18	0.34	0.22	0.46											0.38	0.47	0.00	0.00	1.00	0.05	0.17	0.00	0.00	0.00	0.00	0.00	0.00	0.00	0.00
21-40	S	0.00	0.00	0.00	0.00	0.00	0.44	0.41	0.50	0.00	0.77	0.78	0.26	0.84	0.67	1.00	0.69	0.21	0.69	0.55	0.86	0.75	0.18	0.77	0.66	0.89	0.80	0.09	0.81	0.74	0.85	0.69	0.33	0.79	0.61	1.00	0.63	0.31	0.67	0.48	0.83	0.36	0.26	0.31	0.17	0.51	0.18	0.24	0.02	0.00	0.33	0.00	0.00	0.00	0.00	0.00	0.00	0.00	0.00	0.00	0.00
	N	0.00	0.00	0.00	0.00	0.00	0.09	0.27	0.00	0.00	0.00	0.68	0.44	1.00	0.21	1.00	0.82	0.30	1.00	0.70	1.00	0.80	0.14	0.79	0.74	0.90	0.72	0.12	0.74	0.66	0.82	0.82	0.09	0.82	0.75	0.89	0.49	0.36	0.50	0.19	0.84	0.29	0.38	0.02	0.00	0.58	0.19	0.38	0.00	0.00	0.00	0.09	0.28	0.00	0.00	0.00	0.00	0.00	0.00	0.00	0.00
	F	0.00	0.00	0.00	0.00	0.00	0.15	0.34	0.00	0.00	0.01	0.32	0.37	0.09	0.00	0.51	0.63	0.19	0.59	0.51	0.74	0.49	0.11	0.52	0.45	0.57	0.50	0.07	0.50	0.46	0.56	0.60	0.08	0.61	0.55	0.66											0.00	0.01	0.00	0.00	0.00	0.00	0.00	0.00	0.00	0.00	0.00	0.00	0.00	0.00	0.00
41-60	S	0.00	0.00	0.00	0.00	0.00	0.00	0.00	0.00	0.00	0.00	0.04	0.18	0.00	0.00	0.00	0.30	0.44	0.00	0.00	0.86	0.21	0.40	0.00	0.00	0.13	0.84	0.23	0.93	0.76	1.00	0.80	0.32	0.97	0.75	1.00	0.17	0.36	0.00	0.00	0.00	0.06	0.24	0.00	0.00	0.00	0.05	0.21	0.00	0.00	0.00	0.00	0.00	0.00	0.00	0.00	0.00	0.00	0.00	0.00	0.00
	N	0.21	0.38	0.00	0.00	0.24	0.36	0.43	0.03	0.00	0.82	0.34	0.36	0.19	0.00	0.61	0.58	0.29	0.60	0.43	0.80	0.60	0.28	0.61	0.45	0.84	0.57	0.31	0.55	0.44	0.78	0.79	0.18	0.81	0.71	0.92	0.70	0.23	0.72	0.52	0.91	0.69	0.24	0.70	0.60	0.87	0.47	0.29	0.34	0.25	0.70	0.06	0.13	0.00	0.00	0.00	0.00	0.00	0.00	0.00	0.00
	F	0.21	0.40	0.00	0.00	0.00	0.10	0.22	0.02	0.00	0.03	0.34	0.20	0.35	0.21	0.47	0.52	0.24	0.48	0.37	0.74	0.66	0.28	0.67	0.52	0.92	0.27	0.28	0.14	0.04	0.50	0.73	0.36	0.93	0.50	1.00											0.01	0.08	0.00	0.00	0.00	0.00	0.00	0.00	0.00	0.00	0.00	0.00	0.00	0.00	0.00
61-80	S	0.00	0.00	0.00	0.00	0.00	0.00	0.00	0.00	0.00	0.00	0.00	0.00	0.00	0.00	0.00	0.00	0.00	0.00	0.00	0.00	0.00	0.00	0.00	0.00	0.00	0.00	0.00	0.00	0.00	0.00	0.00	0.00	0.00	0.00	0.00	0.00	0.00	0.00	0.00	0.00	0.00	0.00	0.00	0.00	0.00	0.00	0.00	0.00	0.00	0.00	0.00	0.00	0.00	0.00	0.00	0.00	0.00	0.00	0.00	0.00
	N	0.00	0.00	0.00	0.00	0.00	0.00	0.00	0.00	0.00	0.00	0.00	0.00	0.00	0.00	0.00	0.03	0.17	0.00	0.00	0.00	0.06	0.20	0.00	0.00	0.00	0.07	0.25	0.00	0.00	0.00	0.01	0.08	0.00	0.00	0.00	0.03	0.17	0.00	0.00	0.00	0.00	0.00	0.00	0.00	0.00	0.00	0.00	0.00	0.00	0.00	0.03	0.17	0.00	0.00	0.00	0.00	0.00	0.00	0.00	0.00
	F	0.00	0.00	0.00	0.00	0.00	0.00	0.00	0.00	0.00	0.00	0.06	0.24	0.00	0.00	0.00	0.23	0.32	0.00	0.00	0.50	0.50	0.39	0.50	0.03	0.89	0.55	0.40	0.51	0.17	0.97	0.26	0.40	0.00	0.00	0.68											0.00	0.00	0.00	0.00	0.00	0.00	0.00	0.00	0.00	0.00	0.00	0.00	0.00	0.00	0.00
81-100	S	0.00	0.00	0.00	0.00	0.00	0.00	0.00	0.00	0.00	0.00	0.00	0.00	0.00	0.00	0.00	0.00	0.00	0.00	0.00	0.00	0.00	0.00	0.00	0.00	0.00	0.00	0.00	0.00	0.00	0.00	0.00	0.00	0.00	0.00	0.00	0.00	0.00	0.00	0.00	0.00	0.00	0.00	0.00	0.00	0.00	0.00	0.00	0.00	0.00	0.00	0.00	0.00	0.00	0.00	0.00	0.00	0.00	0.00	0.00	0.00
	N	0.00	0.00	0.00	0.00	0.00	0.00	0.00	0.00	0.00	0.00	0.00	0.00	0.00	0.00	0.00	0.00	0.00	0.00	0.00	0.00	0.00	0.00	0.00	0.00	0.00	0.00	0.00	0.00	0.00	0.00	0.00	0.00	0.00	0.00	0.00	0.00	0.00	0.00	0.00	0.00	0.00	0.00	0.00	0.00	0.00	0.03	0.17	0.00	0.00	0.00	0.00	0.00	0.00	0.00	0.00	0.00	0.00	0.00	0.00	0.00
	F	0.00	0.00	0.00	0.00	0.00	0.00	0.00	0.00	0.00	0.00	0.00	0.00	0.00	0.00	0.00	0.00	0.00	0.00	0.00	0.00	0.00	0.00	0.00	0.00	0.00	0.06	0.24	0.00	0.00	0.00	0.00	0.00	0.00	0.00	0.00											0.00	0.00	0.00	0.00	0.00	0.00	0.00	0.00	0.00	0.00	0.00	0.00	0.00	0.00	0.00

Table S2: Traits and trait states analysed in the present study, description with references, and data sources.

Trait	Trait states	Description	Data source
Life span	Annual; biennial; perennial	Life span of each species	1, 2; checked and supplemented by field observations
Vegetative propagation	Absent; bulbils; rhizome; root, tuber or bulb splitter; runner/runner-like rhizome	Occurrence and type of vegetative propagation, identified following Krumbiegel[3], and Klimešová and de Bello[4]	2, 5, checked and supplemented by field observations
Storage organ	Absent; bulb; persistent tap root; rhizome; tuber	Occurrence and type of storage organ identified following Krumbiegel[3] and Klimešová and de Bello[4]	2, 5, checked and supplemented by field observations
Leaf persistence	Overwintering green leaves; persistent green leaves; spring green leaves; summer green leaves	Classification of how long a leaf persists on a plant from emergence until cast, according the categories indicated in Klotz and Kühn[6]	2; checked and supplemented by field observations made during the year
Leaf anatomy	Hygromorphic; mesomorphic; scleromorphic; succulent	Main structures within the leaves to fulfil specific tasks (e.g., aeration, supporting tissues, water storage), identified following Klotz and Kühn[6] and Küster et al.[7]. Species may have more than one type of leaf anatomy (e.g. scleromorphic/ mesomorphic; mesomorphic/ hygromorphic). Each combination of leaf anatomy types was considered in statistical elaborations.	2; checked and supplemented by the authors' observations
Horizontal space occupation	Absent; caespitose; climber; pleiocorm; prostrate; reptant; rosulate	Classification of horizontal growth form, according to the categories indicated in Krumbiegel[3]	1, 2; checked and supplemented by the authors' observations
Vertical space occupation	Climber; erosulate upright forb; hemirosulate upright forb; leafy stem, narrow leaves (grass); leafy stem prostrate on the ground (prostrate forb); no leafy stem, with broad basal leaves (rosette forb); no leafy stem, with narrow basal leaves	Classification based on the width of leaves and on their position along the stem[3,8]	1, 2; checked and supplemented by the authors' observations
Plant height (cm)	≤ 20; 21-40; 41-60; 61-80; 81-100	Plant height categorized into five classes of the same amplitude. To have a range of variation for each species, we analyzed specimens collected in the study area in June, when plants reach their maximum size. The entire range of plant heights was divided into five 20 cm wide classes, deemed wide enough to encompass a large part of the range of intra-specific variability of each species, and, at the same time, to limit the number of categories. Indeed, the more categories chosen, the higher the potential dissimilarity for a given trait[9]. We attributed each species to the class that included most of its range of variability (at least 70%).	Field measurements and measurements taken on specimens stored in the *Herbarium* of the University of Camerino (CAME)

References

1. Pignatti, S. *Flora d'Italia* [*Flora of Italy*] (Edagricole, 1982).
2. Klotz, S., Kühn, I. & Durka, W. Biolflor: search and information system on vascular plants in Germany http://www2.ufz.de/biolflor/index.jsp Accessed on 15 June 2016
3. Krumbiegel, A. Morphologie der vegetative Organe (auser Blatter) [*Morphology of vegetative organs (except leaves)*] in *Biolflor: Eine Datenbank zu biologisch-ökologischen Merkmalen der Gefäßpflanzen in Deutschland* [*BiolFlor: A database on bio-ecological characteristics of vascular plants*] (eds. Klotz, S., Kühn, I. & Durka, W.) 93–118 (Bundesamt for Naturschutz, 2002).

4. Klimešová, J. & de Bello, F. Clo-Pla: the database of clonal and bud bank traits of Central European flora. *J. Veg. Sci.* **20,** 511–516 (2009).
5. Klimešová, J. & Klimeš, L. Clo-Pla3 - database of clonal growth of plants from Central European. http://clopla.butbn.cas.cz Accessed 15 June 2016
6. Klotz, S. & Kühn, I. Blattmerkmale [Leaf traits] in *Biolflor*: *Eine Datenbank zu biologisch-ökologischen Merkmalen der Gefäßpflanzen in Deutschland* [*BiolFlor: A database on bio-ecological characteristics of vascular plants*] (eds. Klotz, S., Kühn, I. & Durka, W.) 119–126 (Bundesamt for Naturschutz, 2002).
7. Küster, E.C., Bierman, S.M., Klotz, S. & Kühn, I. Modelling the impact of climate and land use change on the geographical distribution of leaf anatomy in a temperate flora. *Ecogr.* **33,** 1-12 (2010).
8. Liira, J. & Zobel, K. Vertical structure of a species-rich grassland canopy, treated with additional illumination, fertilization and mowing. *Plant Ecol.* **146,** 185–195 (2000).
9. de Bello, F., Lepš, J. & Sebastià, M.T. Variations in species and functional plant diversity along climatic and grazing gradients. *Ecogr.* **29,** 801–810 (2006).

Figure S1: Trends of mean monthly temperature and precipitation during the 2011 growing season.

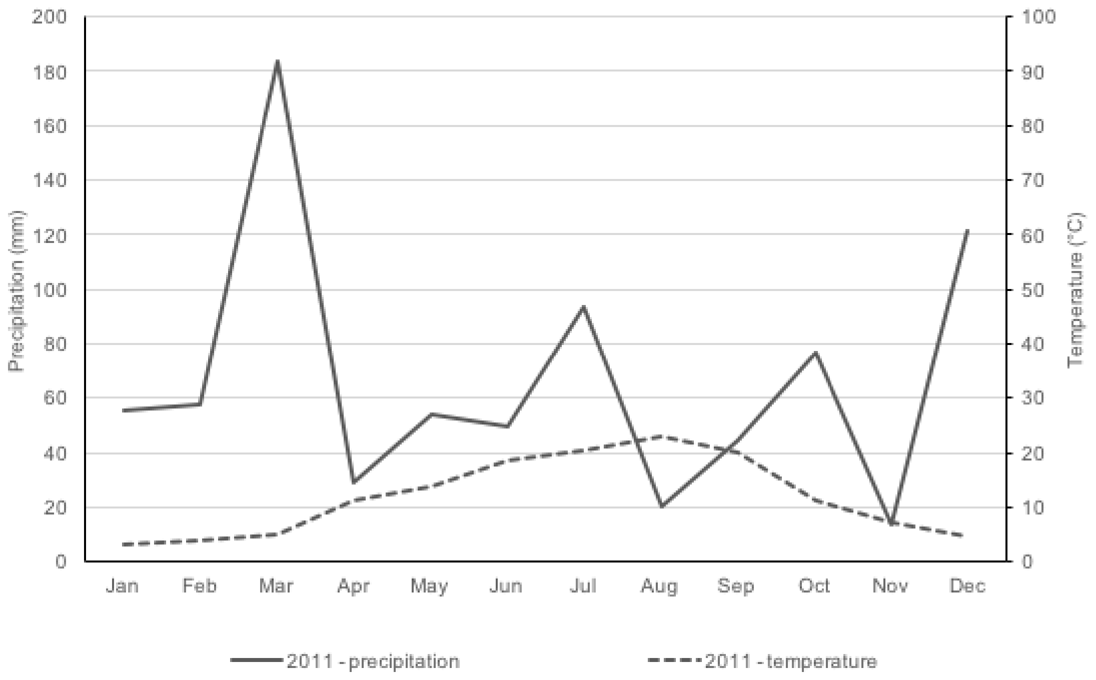

Figure S2: Trends of average soil temperature (a), soil relative humidity (b) and canopy height (c) in grassland communities located on south-facing slopes, north-facing slopes, and flat lands, during the 2011 growing season. 1 - 4 Apr; 2 - 18 Apr; 3 - 2 May; 4 - 16 May; 5 - 30 May; 6 - 13 Jun; 7 - 30 Jun; 8 - 20 Jul; 9 - 5 Aug; 10 - 19 Aug; 11 - 6 Sep; 12 - 21 Oct.

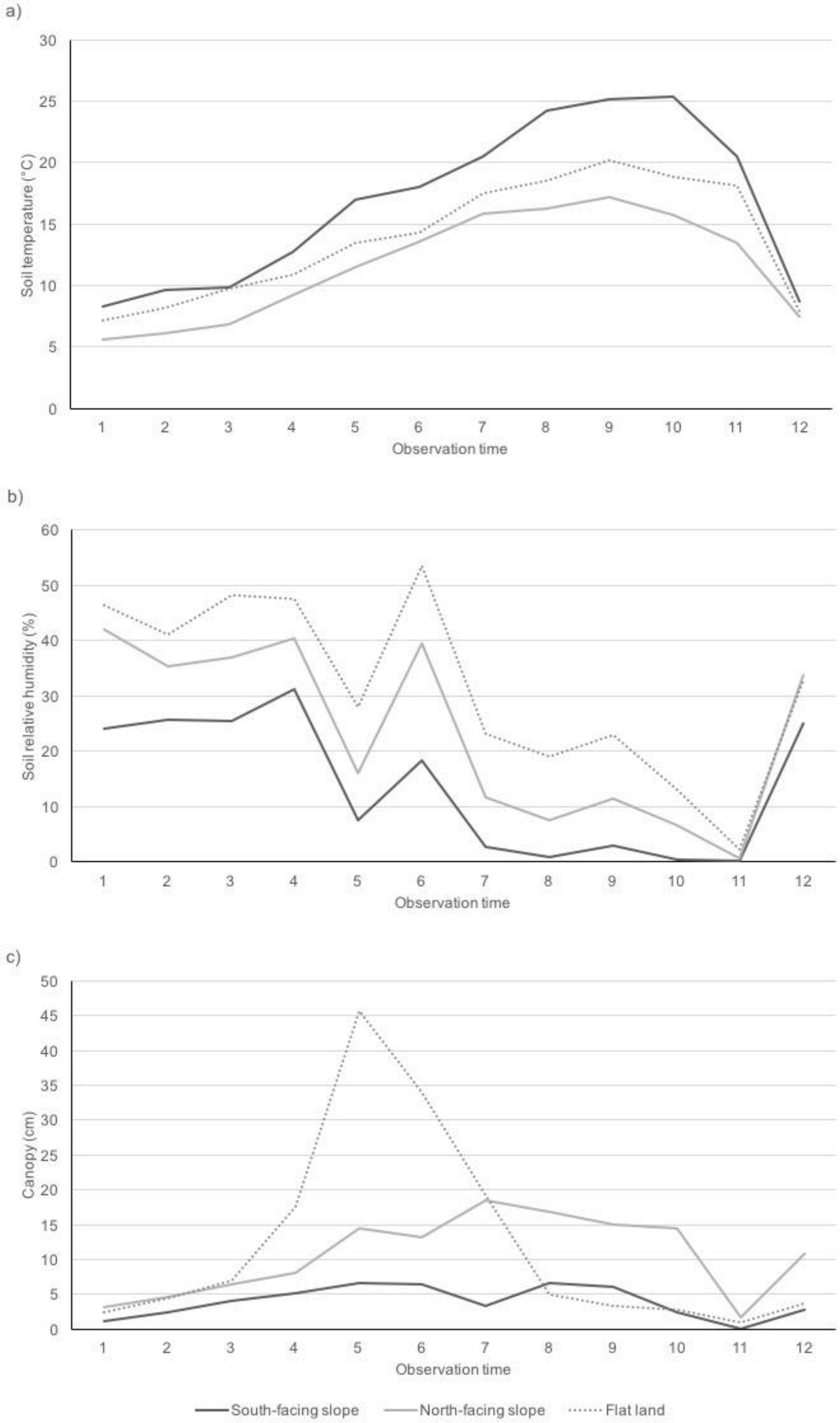

Supplement: Supplementary file 1 — Supporting information [file 41598_2017_12226_MOESM1_ESM.pdf]
